# Modeling of Kidney Hemodynamics: Probability-Based Topology of an Arterial Network

**DOI:** 10.1371/journal.pcbi.1004922

**Published:** 2016-07-22

**Authors:** Dmitry D. Postnov, Donald J. Marsh, Dmitry E. Postnov, Thomas H. Braunstein, Niels-Henrik Holstein-Rathlou, Erik A. Martens, Olga Sosnovtseva

**Affiliations:** 1 Department of Biomedical Sciences, University of Copenhagen, Copenhagen, Denmark; 2 Department of Molecular Pharmacology, Physiology, and Biotechnology, Brown University, Providence, Rhode Island; 3 Physics Department, Saratov State University, Saratov, Russian Federation; University of Michigan, UNITED STATES

## Abstract

Through regulation of the extracellular fluid volume, the kidneys provide important long-term regulation of blood pressure. At the level of the individual functional unit (the nephron), pressure and flow control involves two different mechanisms that both produce oscillations. The nephrons are arranged in a complex branching structure that delivers blood to each nephron and, at the same time, provides a basis for an interaction between adjacent nephrons. The functional consequences of this interaction are not understood, and at present it is not possible to address this question experimentally. We provide experimental data and a new modeling approach to clarify this problem. To resolve details of microvascular structure, we collected 3D data from more than 150 afferent arterioles in an optically cleared rat kidney. Using these results together with published micro-computed tomography (*μ*CT) data we develop an algorithm for generating the renal arterial network. We then introduce a mathematical model describing blood flow dynamics and nephron to nephron interaction in the network. The model includes an implementation of electrical signal propagation along a vascular wall. Simulation results show that the renal arterial architecture plays an important role in maintaining adequate pressure levels and the self-sustained dynamics of nephrons.

## Author Summary

By maintaining the volume and composition of the body fluids within narrow ranges, and by producing a set of hormones that affect the blood vessels, the kidneys provide important long-term regulation of blood pressure. Disturbances of kidney function can cause hypertension, a prevalent disease in modern societies. The kidneys protect their own function against short-term variations in blood pressure at the level of the individual unit (the nephron). In recent years, it has become clear that there is an interaction between nephrons, and that this interaction is mediated through the arterial network of the kidney. The renal vacular network has a complex topology, and at present there are no computational models of this topology, precluding a computational assessment of the consequences of nephron-nephron interactions for renal blood flow control. In this work we focus on understanding how kidney specific vascular structure affects blood flow patterns and nephron-to-nephron interaction in kidney. The paper presents an approach to constructing realistic models of the renal vascular architecture. We developed a computational approach to reproduce the architecture and to examine its consequences for the operating regime of the nephrons.

## Introduction

An important feature of the vascular system is its highly branched structure. It is assumed that the branching geometry of blood vessels is governed by defined principles [1–3]. Tree-like structures of arterial systems have been a subject of experimental and theoretical studies related to vascular topology and flow [4–6]. Structural connectivity in vascular networks directly influences their functional state, defining how much oxygen and nutrients are delivered to different regions of the organism. The network experiences local responses affecting the local blood flow and altering the overall state of the network [7–9]. The topology of the vascular network is also an important determinant of the consequences of pathological processes in the vessels [10].

Nephrons are arranged in a complex branching structure, the nephro-arterial network, that delivers blood to each nephron and, at the same time, provides the basis for an interaction between each nephron and neighboring ones. The interaction between nephrons across the arterial network results in complex spatial patterns of synchronization between tubular pressure oscillations of the individual nephrons. This was demonstrated in several recent studies employing measurements of renal cortical blood flow using laser speckle flowmetry [11–14]. At present, both the functional significance of spatial patterns and the mechanisms leading to their formation are unknown.

An important step in the investigation of the mechanisms and the functional significance of the spatial synchronization of cortical blood flow is a better understanding of the topology of the renal circulation. This requires a detailed mapping of the entire renal vascular tree, a task that has become feasible due to the development of micro-computed tomography (*μ*CT) [15] and optical clearance methods [16, 17]. Such data provide a statistical basis from which computational models of the renal circulation can be generated.

Two main features of the nephron-vascular network are (i) a significant pressure drop between the origin of an afferent arteriole and its insertion into the glomerulus, and (ii) the presence of the tubulo-glomerular feedback mechanism. Experimental results [18, 19] show that neighboring nephrons adjust their TGF-mediated tubular pressure oscillations to attain a synchronized regime.Neighboring nephrons interact by vascular propagation of electrical signals, initiated by the TGF of each nephron, which travel via endothelial cells in the arteriolar wall and can reach (the branch point with) the neighboring afferent arteriole. This coupling tends to produce simultaneous constriction of the afferent arterioles leading to in-phase synchronization of the oscillations. Another type of interaction, hemodynamic coupling, occurs due to the topology of the renal vasculature. Sharing of blood flow at each branch point of the arterial tree provides an interdependence of the local perfusion pressures, and, hence, affects the hemodynamics of all nephrons located downstream of the branch point. Due to a significant time lag in the system, this type of interaction can lead to anti-phase synchronization betweeen nephrons. Depending on the precise topology of the arteriolar network the first of these mechanisms may be more important for the local coupling of nephrons, with the other mechanism more important for global coupling.

Several recent modeling studies have investigated features of nephron-nephron interactions [20–22]. The complex renal arterial network in these studies was approximated by a simple bifurcating tree-like structure and included a group of 10–20 nephrons. Postnov et al. [20] showed that such a topology induces desynchronization between originally identical nephron models (coupling-induced inhomogeneity). Marsh et al. [21] demonstrated the presence of steady state, quasiperiodic and chaotic dynamics in an ensemble of cortical and medullary nephrons depending on the interaction strengths and the arterial blood pressure. Bayram et al. [22] suggested that several nephrons originating from the same small artery are more likely to be in an oscillatory state than a single nephron will. We recently demonstrated [23] that (i) neighboring nephrons at the same branch level were synchronized in-phase due to the vascular propagated electrical coupling, (ii) nephrons separated by one branch point tended to display a phase-shifted pattern due to hemodynamic coupling, and (iii) distantly located groups of nephrons showed asynchronous behavior. Marsh et al. [24] introduced heterogeneity into the nephro-arterial network, and observed similar dynamical patterns.

However, all of the studies cited used an overly simplified representation of the renal arterial network, and it is not clear to what extent the observed dynamics might be a consequence of the simplification. Further progress requires the use of more realistic models of the renal arterial network. In this study we focus on understanding how renal arterial structure affects blood flow dynamics. To address this question: (i) we perform experiments using optical tissue clearance to resolve details of microvascular structure not previously available; (ii) we develop an algorithm for generating a realistic model of the renal arterial network using the data obtained from our experiments and from micro-computed tomography study by Nordsletten et al [15]; (iii) we present a mathematical model that describes blood flow dynamics and nephron to nephron interaction in the renal nephro-arterial network including a new implementation of electrical signal propagation along a vascular wall; and (iv) we simulate how renal specific vascular structure can affect renal blood flow patterns and nephron-to-nephron interactions.

## Model

### Algorithm for the vascular structure

We suggest a general algorithm to create an asymmetrical bifurcating tree based on experimental data, with modifications that are specific for the renal microcirculation:

- An asymmetric bifurcating tree (ABT) with a gradual decrease of diameter, and with afferent arterioles present only at the end of the tree;
- A kidney specific asymmetric bifurcating tree (KSABT) with a fraction of afferent arterioles branching directly from larger vessels.

**Fig 1:**
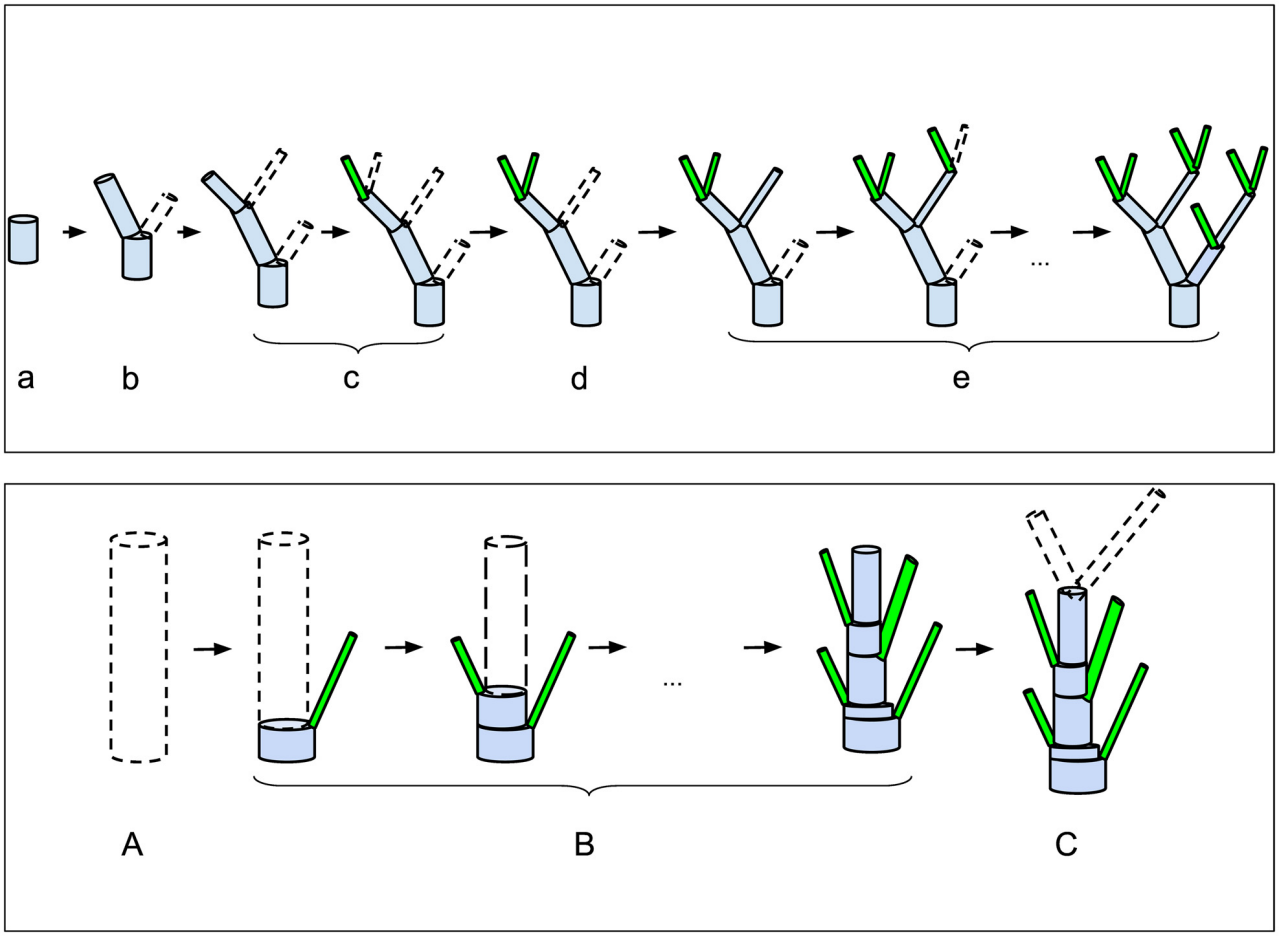
Bifurcation algorithms. Top panel: Main steps of the recursive bifurcating algorithm to create an ABT. Starting with a vessel of a certain diameter, (a) determine the vessel length in accordance with its diameter; (b) estimate diameters and lengths of daughter vessels; (c) apply (b) to the first daughter vessel and continue recursively until *D*_*stop*_ is reached; (d) apply (b) to the last “unprocessed” daughter vessel; (e) apply (b), (c), and (d) until the whole tree is built. Bottom panel: Additional segmentation for kidney specific structure KSABT. (A) Determine initial diameter and length of the vessel; (B) Grow afferent arterioles (instead of daughter vessels); this is accompanied by the diameter reduction of the initial vessel in accordance with Murray’s law; (C) after the entire length of the vessel is processed, estimate the diameter of daughter vessels based on the new (reduced) diameter of the current vessel. Except segmentation, other steps of creating KSABT are the same as of ABT. Detailed description of the algorithm can be found in Materials and Methods.

In both cases the structure of the vascular networks is simulated using a probability-based bifurcating algorithm together with Murray’s law [1]. Main features and assumptions of the suggested algorithm are described below:

1. Simulations start from a vessel with the initial diameter *D*_*initial*_.
2. A vessel always bifurcates into two daughter vessels;
3. For a daughter vessel the distribution of its diameter is a function of the parent vessel diameter;
4. The diameter of the second branching daughter vessel is calculated according to Murray’s law [1], i.e. $D_{d2}=\sqrt[{3}]{D_{p}^{3}-D_{d1}^{3}}$, where *D*_*p*_, *D*_*d*1_, *D*_*d*2_ denote diameters of the parent vessel, the first and second daughter vessels, respectively (see algorithm visualization in Fig 1, top panel);
5. The distribution of vessel lengths is a function of the vessel diameter;
6. Vessels bifurcate until they reach a given diameter (parameter *D*_*stop*_). Vessels with a diameter less than *D*_*stop*_ are counted as afferent arterioles;
7. (Only for KSABT). Additional afferent arterioles are distributed over the whole vascular tree and appear with a probability according to the distribution of distances between neighboring afferent arterioles. We use a segmentation algorithm for this step. (Fig 1, bottom panel). Murray’s law is still applied at each branch point;
8. (Only for KSABT). If the first vessel is the renal artery, it is not segmented and can not have afferent arterioles. Otherwise segmentation is applied.

A more detailed description can be found in Materials and Methods.

### Statistical data on network topology

**Fig 2:**
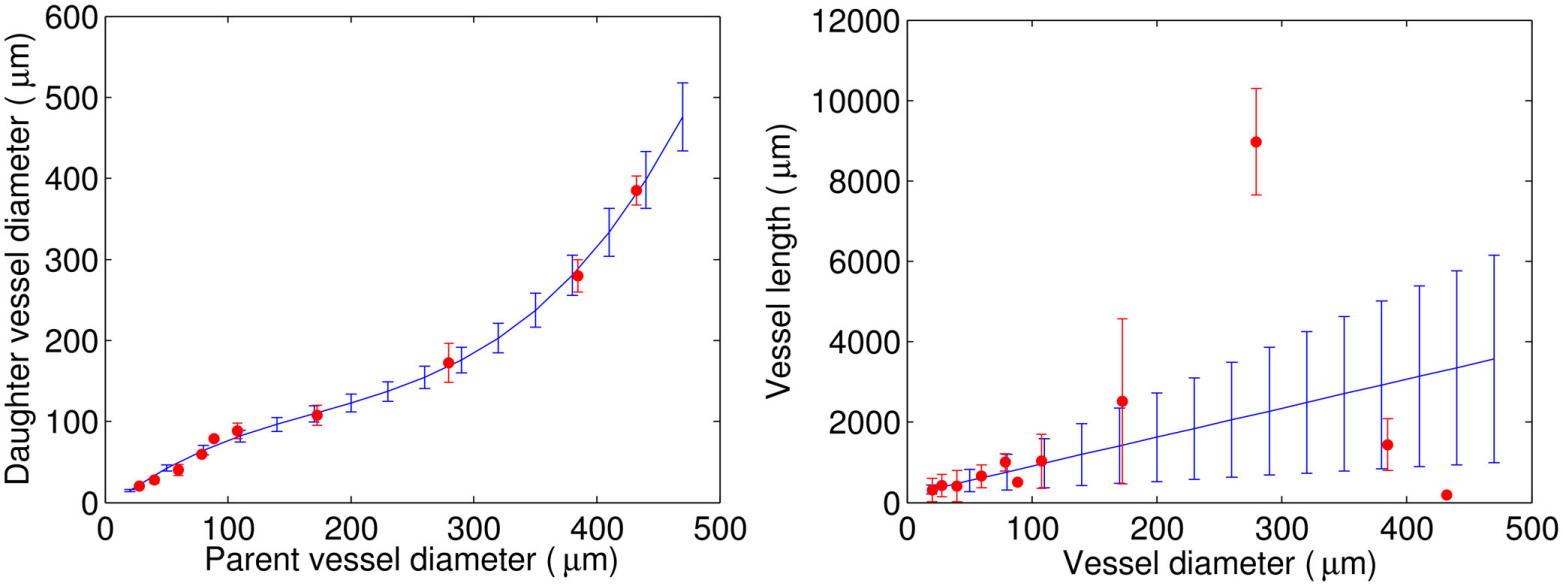
Distributions based on the data published by Nordsletten et al. [15] (red) and their approximations (blue). Left panel: Daughter vessel diameter as a function of the parent vessel diameter. Right panel: Vessel length as a function of the vessel diameter.

The available data were collected by means of *μ*CT with 20-*μ*m and 4-*μ*m resolution and published by Nordsletten et al. [15]. For the distribution of daughter vessel diameters as a function of the parent vessel diameter we use a cubic approximation for mean values and a linear approximation for standard deviations (Fig 2, left panel). For simplicity, it is assumed that branch level corresponds to a Strahler order. For a given parent vessel diameter, we use the cubic approximation to find the mean and standard deviation for the distribution of daughter vessel diameters. The diameter for the first daughter vessel is then drawn at random from this distribution. The diameter of the second daughter vessel is calculated so that it complies with Murray’s law. For the length distribution as a function of the vessel diameter, a linear approximation is used for both mean and standard deviations for the best fit in the range of [20;100] *μ*m (Fig 2, right panel). Based on these approximations we build a vascular tree with gradually decreasing diameter and with afferent arterioles located only at the end of the tree (ABT). Nordsletten et al. found only a small deviation (≈1%) between a regression analysis performed on all daughter-parent diameter relationships and Murray’s law [15], a finding that justifies our use of it. Accordingly, we designed our algorithm to ensure that the vascular tree it generates also follows Murray’s law. Because vessel diameters at a bifurcation fulfill Murray’s law, they are not statistically independent. This reflects the fact that knowing the diameters of two of the three vessels in a bifurcation, the diameter of the last vessel can be calculated from Murray’s law. It should also be noted that the assumption that Strahler order corresponds to branching order may introduce some errors. Two daughter vessels need not have the same Strahler order, as this depends on the number of bifurcations that occur downstream from the respective daughter vessels [25–27]. Thus, the Strahler ordering scheme categorizes the vessels starting from the smallest vessels going upstream to the larger vessels. In principle it is therefore not possible to assign a Strahler order to a given vessel before the downstream branching process has come to an end. Since our algorithm “works” from the larger vessels towards the smaller ones, the Strahler ordering scheme is incompatible with the bifurcating algorithm, since it, in a sense, only can be applied retrospectively. However, the effect of this is expected only to be of minor significance.

The data obtained by *μ*CT provide important information about the dimensions of the larger renal vessels. However, due to the limited spatial resolution, important information on afferent arterioles is missing. Different studies show that (i) afferent arterioles (AA) can branch not only from small vessels, but also from larger arteries [28, 29] and (ii) two, three, or four afferent arterioles can originate together from a single site on an artery [30]. These features make the renal vascular structure significantly different from the vascular structure of other organs [31, 32].

We assume that the distances between the origins of AAs on the vascular tree can be described by an exponential distribution. This assumption is based on several observations:

1. AAs often originate in doublets or triplets even from large vessels. This implies a high probability of zero or close to zero distances between neighboring AAs;
2. Vessel segments without AAs branching from them are rarely seen. This implies a low probability for distances between AAs of several hundreds of microns.

The simplest possible distribution that satisfies observations 1 and 2 is an exponential distribution. Finally, we assumed that the distribution of the distance between neighboring AAs is independent of the diameter of the feeding vessel.

**Fig 3:**
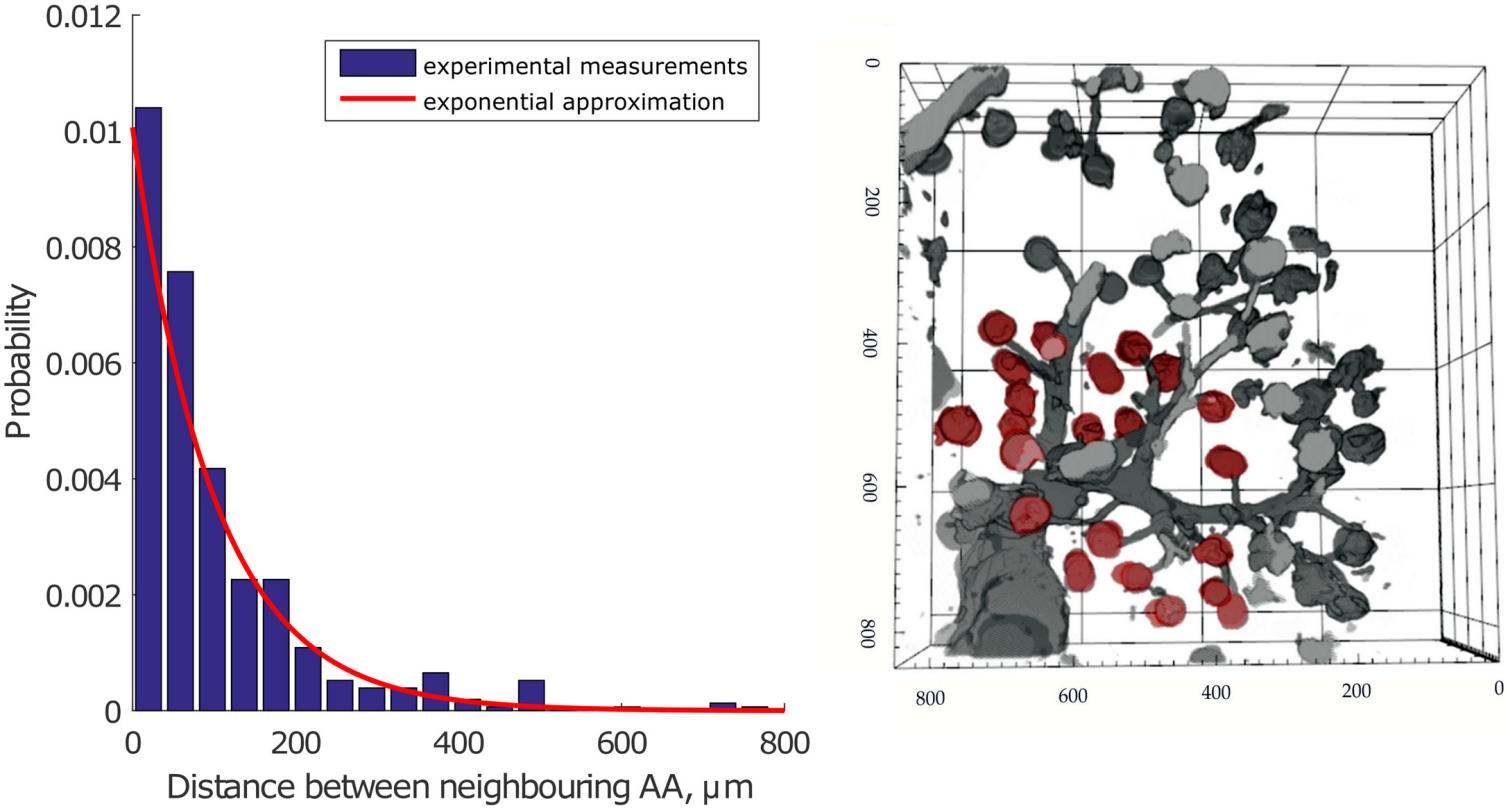
Left panel: Measurements of the distance between neighboring AAs based on the data from cleared renal tissue (blue) and the exponential approximation (red). Experimental data are normalized to the area under the distribution. Right panel: Reconstructed image shows a stack of renal vessels. Afferent arterioles branch from large vessels that continue their branching (glomeruli are depicted in red color). The dimensions of the stack are 850 x 850 x 211 *μ*m. Axis labels show the original coordinates in *μ*m, but note that the tissue shrinks ≈ 20% in each direction during the optical clearing procedure (see Material and Methods). A correction factor for tissue shrinkage was included into the measurements in the left panel.

To provide experimental support for using an exponential distribution for the distances between the origins of afferent arterioles, we performed experimental studies to visualize renal vascular architecture using confocal microscopic imaging of chemically cleared renal tissue (see details in Materials and Methods) [16, 17]. We obtained two 3D stacks of renal vascular structure, from which we measured the distances between the origins of neighboring AAs. An example of the observed structures is shown in Fig 3, right panel. The data set contains information on distances between origins of more than 150 afferent arterioles branching from the feeding vessels with diameters of 25–100 *μ*m. An exponential function (red) provides a good fit to the data (blue) (Fig 3, left panel). The exponential distribution is used to generate a vascular tree specific to the kidney (KSABT) where afferent arterioles may branch off any vessel on the arterial structure.

Using the algorithm described above and the statistical data on renal vascular morphology, a renal vascular structure is generated in the form of a connectivity matrix that maps the connections between vessels as well as the vessel length and diameter. The matrix is used as the input for full-scale computer simulations.

### Hemodynamic interaction

A hemodynamic interaction occurs when one nephron is stimulated by its TGF-mechanism to contract its afferent arteriole, causing the hydrostatic pressure to rise at the upstream branch node, increasing the blood flow to the second nephron. Half a period later when the increased blood flow activates the TGF-mechanism in the second nephron and causes its afferent arteriole to contract, the blood flow to this nephron is reduced, and the blood flow to the first nephron increases. Due to the time lag in the response, this type of interaction tends to induce anti-phase synchronization between nephrons.

To describe the flow in each vessel of the vascular tree, one needs the hemodynamic resistance *R*_*hdr*_ calculated from the Poiseuille relationship:

$$R_{hdr}=\frac{128\eta L}{\pi D^{4}}.$$ (1)

Here *L* is the length of the vessel, *η* is the viscosity of blood. The viscosity depends on vessel diameter, and we adopt the expression for viscosity from Ref. [33], assuming an invariant hematocrit of 0.45:

$$\eta =\left(1+\left(6exp\left(-0.085D\right)+2.2-2.44exp\left(-0.06D^{0.645}\right)\right){\left(\frac{D}{D-1.1}\right)}^{2}\right){\left(\frac{D}{D-1.1}\right)}^{2},$$ (2)

where *η* is in units of centipose and *D* is the inner diameter of the vessel in micrometers.

**Fig 4:**
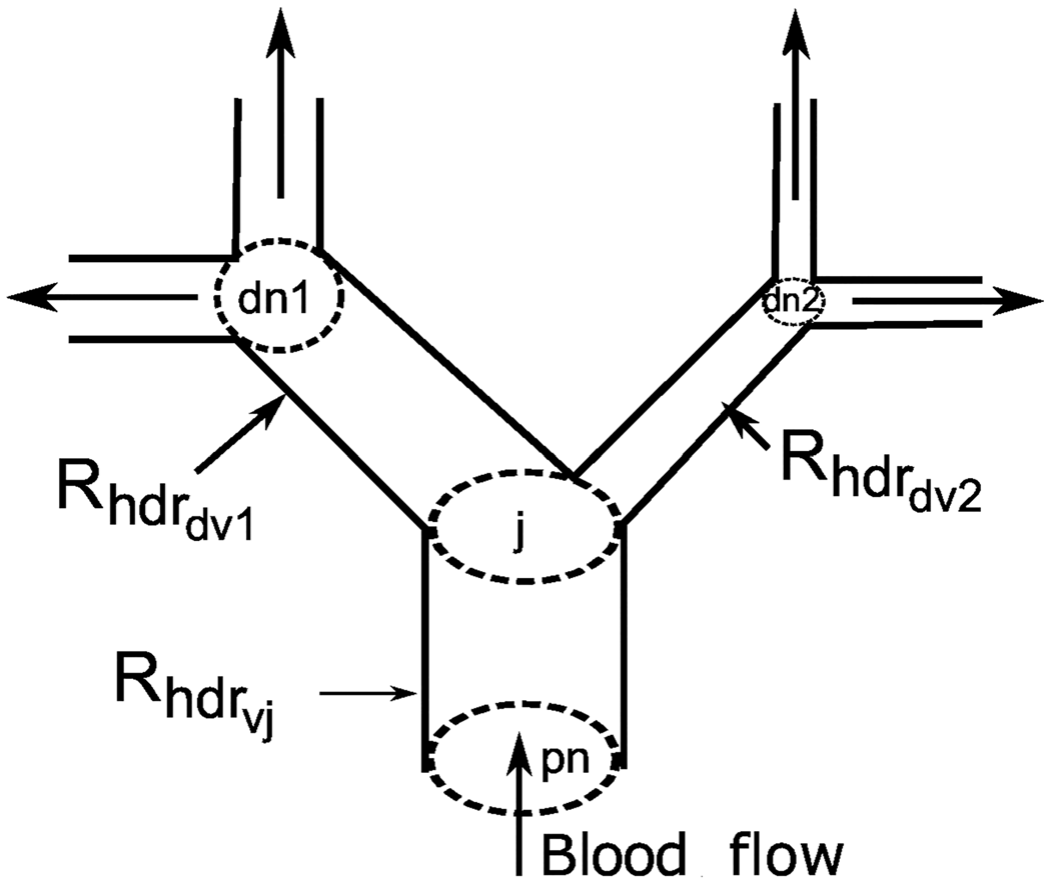
A fragment of the vascular structure. *pn*, *dn*1, *dn*2 and *j* denote the parent node, the first daughter node, the second daughter node, and the current node, respectively. Each vessel has its own hemodynamic resistance.

The pressure variation in each node, derived from expressions for conservation of flow in each node (Fig 4), is given by:

$$\text{afferent}:\;\;C_{hdr}\frac{dP_{j}}{dt}=\frac{P_{pn_{j}}-P_{j}}{R_{hdr_{v_{j}}}}-F_{neph_{j}},$$ (3)

$$\text{other}:\;\;C_{hdr}\frac{dP_{j}}{dt}=\frac{P_{pn_{j}}-P_{j}}{R_{hdr_{v_{j}}}}-\frac{P_{j}-P_{dn1_{j}}}{R_{hdr_{dv1_{j}}}}-\frac{P_{j}-P_{dn2_{j}}}{R_{hdr_{dv2_{j}}}}.$$ (4)

Here *C*_*hdr*_ is the vessel compliance. *P*_*pn*_*j*__, *P*_*dn*1_*j*__, *P*_*dn*2_*j*__, and *P*_*j*_ denote pressure in the parent node, the first daughter node, the second daughter node, and the current node, respectively. *R*_*hdr*_*v*_*j*___, *R*_*hdr*_*dv*1_*j*___, and *R*_*hdr*_*dv*2_*j*___ are the hemodynamic resistance of the current vessel, the first daughter vessel and the second daughter vessel, respectively. Pressure in the root of the tree and blood flow to the nephrons *F*_*neph*_*j*__ serve as boundary conditions, where *F*_*neph*_*j*__ is calculated for each nephron as the difference between the pressure in the node giving rise to the afferent arteriole and the glomerular capillary pressure divided by the hemodynamic resistance of the afferent arteriole. The hemodynamic resistance is determined from the nephron model (Eq (16).

### Electrical interaction

**Fig 5:**
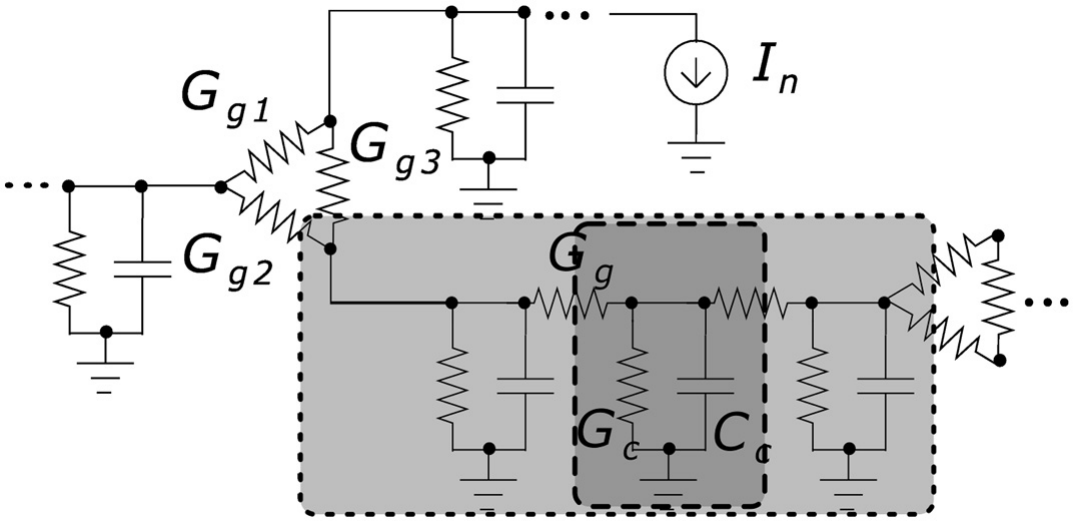
Equivalent electrical circuit of a vessel segment (light gray) located between two branch points. Branch point conductance is modeled as triangle-shaped coupling *G*_*g*1_–*G*_*g*2_–*G*_*g*3_. A vessel segment consists of several units (dark gray) whose length is equal to the length of an endothelial cell with the conductance *G*_*c*_ and the capacitance *C*_*c*_. Units are coupled via gap junctions with conductance *G*_*g*_. Details are given in Materials and Methods.

Activation of TGF in one nephron causes membrane depolarization and contraction of the vascular smooth muscle cells of the corresponding afferent arteriole. Because the cells of the renal vessels are coupled electrically by gap junctions [34], the membrane depolarization spreads, through electrotonic conduction, into the vascular smooth cells of the neighboring AAs causing them to contract [35]. Thus, the cells of the renal vasculature constitute a well-coupled syncytium in which the endothelial cells form the path of least resistance [36]. The deflection in the membrane potential generated by the *macula densa* decays exponentially with the distance between the site of contact between the *macula densa* and the AA [35, 37]. The electrotonic spread between the vascular cells is fast, and it acts to adjust the TGF-mediated tubular pressure oscillations to attain a state of in-phase synchronization [18]. Fig 5 shows a schematic representation of the electrical coupling.

Electrotonic propagation of the electric current originating from the *macula densa* through vascular segments is described by the following equations:

For segment sites that are connected to nephrons:

$$C_{u}\frac{dV}{dt}=I_{n}+G_{g}\left(V_{prev}-V\right)-G_{u}\left(V-V_{rest}\right),$$ (5)

where *I*_*n*_ stands for the current induced by the *macula densa* signal and transmitted to the vessel. *V*_*prev*_ describes the voltage in the previous (downstream) adjacent unit of the same segment. *V*_*rest*_ is the resting membrane potential, *C*_*u*_ is the unit capacitance. *G*_*u*_ and *G*_*g*_ are the unit and gap junction conductance, respectively.

For inner units of the segment:

$$C_{u}\frac{dV}{dt}=G_{u}\left(V_{prev}+V_{next}-2V\right)-G_{u}\left(V-V_{rest}\right),$$ (6)

where *V*_*prev*_ and *V*_*next*_ denote potentials for downstream and upstream adjacent units, respectively.

At the branch point (downstream of the vessel):

$$C_{u}\frac{dV}{dt}=G_{g1}\left(V_{1}-V\right)+G_{g2}\left(V_{2}-V\right)+G_{g}\left(V_{next}-V\right)-G_{u}\left(V-V_{rest}\right),$$ (7)

where *V*_1,2_, and *G*_*g*1,2_ represent the voltages at the ends of connected vessel segments and gap junction conductances, respectively. If the branch point is upstream relative to the current segment then *V*_*prev*_ should be used instead of *V*_*next*_. If the current segment has branch points from both sides then *G*_*g*_(*V*_*next*_ − *V*) should be replaced with the corresponding conductances and voltages for the connected vessels. For the root segment *G*_*g*1_(*V*_1_ − *V*) + *G*_*g*2_(*V*_2_ − *V*) should be replaced with specific values for *G* and resting values for *V*_1_ and *V*_2_.

### Nephron model

**Fig 6:**
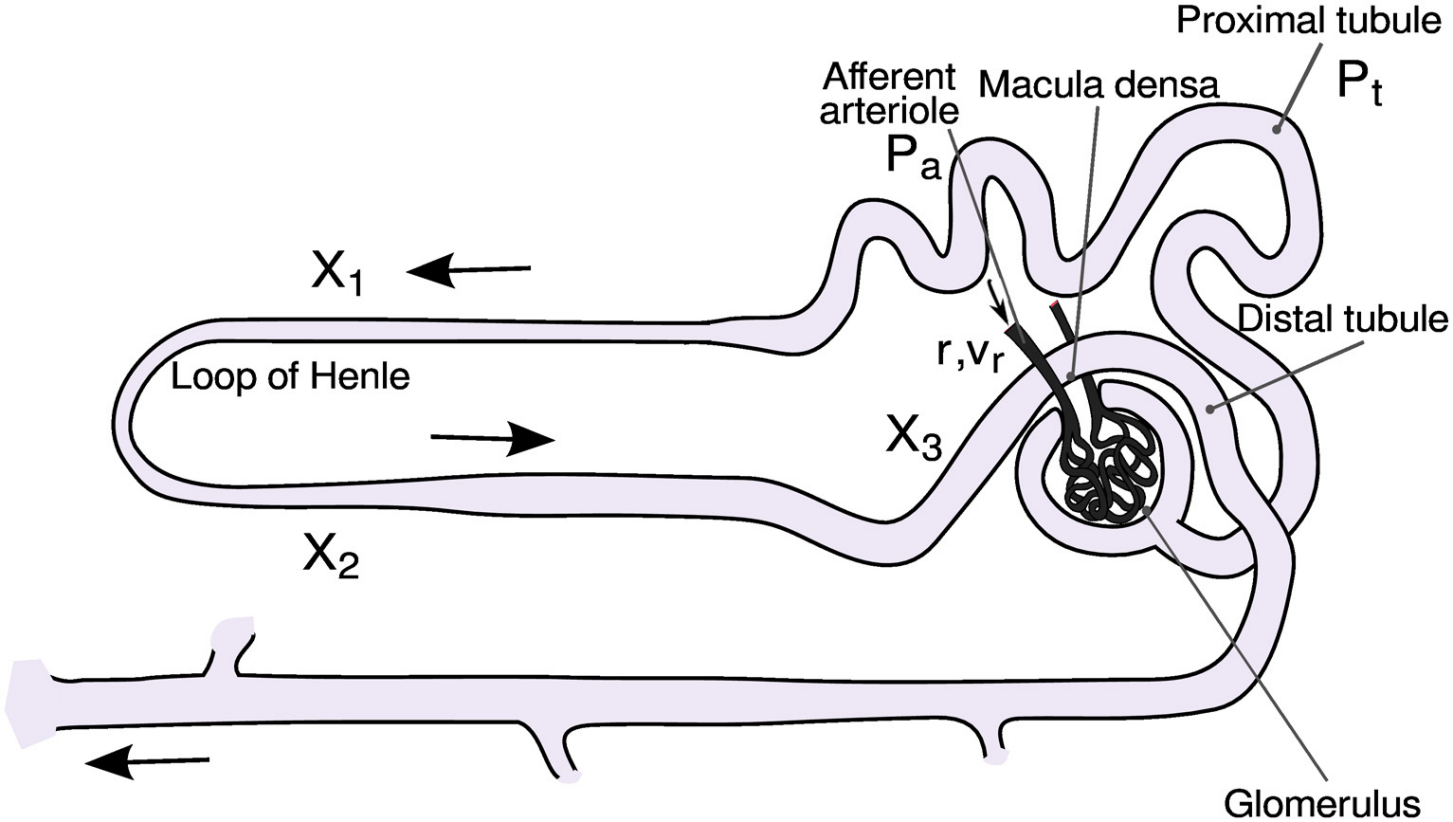
Sketch of the main components of the nephron. Note particularly how the terminal part of the loop of Henle passes within cellular distances of the afferent arteriole, allowing the TGF mechanism to control the incoming blood flow in response to the ionic composition of the fluid leaving the loop of Henle.

The model of the individual nephron consists of six coupled ordinary differential equations, each representing an essential physiological relation and a number of algebraic functions. A sketch of the main components of the nephron model is given in Fig 6. The six coupled differential equations are (see Material and Methods, [38] for details):

$$\frac{dP_{t}}{dt}=\left[F_{filt}-F_{reab}-F_{Hen}\right]/C_{tub}$$ (8)

$$\frac{dr}{dt}=v_{r}$$ (9)

$$\frac{dv_{r}}{dt}=\frac{P_{av}-P_{eq}}{\omega }-K\times v_{r}$$ (10)

$$\frac{dX_{1}}{dt}=F_{Hen}-\frac{3}{T}\times X_{1}$$ (11)

$$\frac{dX_{2}}{dt}=\frac{3}{T}\times \left(X_{1}-X_{2}\right)$$ (12)

$$\frac{dX_{3}}{dt}=\frac{3}{T}\times \left(X_{2}-X_{3}\right)$$ (13)

The first equation of the model (eq 8) represents the pressure variations in the proximal tubule in terms of the in- and outgoing fluid flows. Here, *F*_*filt*_ is the single-nephron glomerular filtration rate and *C*_*tub*_ is the elastic compliance of the tubule. The flow into the loop of Henle is determined by the difference (*P*_*t*_ − *P*_*d*_) between the proximal and the distal tubular pressures and by the flow resistance *R*_*Henle*_. The reabsorption in the proximal tubule *F*_*reab*_ is assumed to be constant.

Eqs 9 and 10 describe the dynamics of the afferent arteriole. Here, *r* represents the inner radius of the vessel and *v* is its rate of change. *ω* is a measure of the mass relative to the elastic compliance of the arteriolar wall, *P*_*av*_ denotes the average pressure in the arteriole, and *P*_*eq*_ is the value of this pressure for which the arteriole is in equilibrium with its present radius and level of muscular activation. The expressions for *F*_*filt*_, *P*_*av*_ and *P*_*eq*_ involve a number of algebraic equations that must be solved along with the integration.

The remaining equations (eqs 11–13) in the single-nephron model describe the delay *T* in the TGF regulation. This delay arises both from the transit time through the loop of Henle and from the cascaded enzymatic processes between the macula densa cells and the smooth muscle cells that control the contraction of the afferent arteriole.

**Fig 7:**
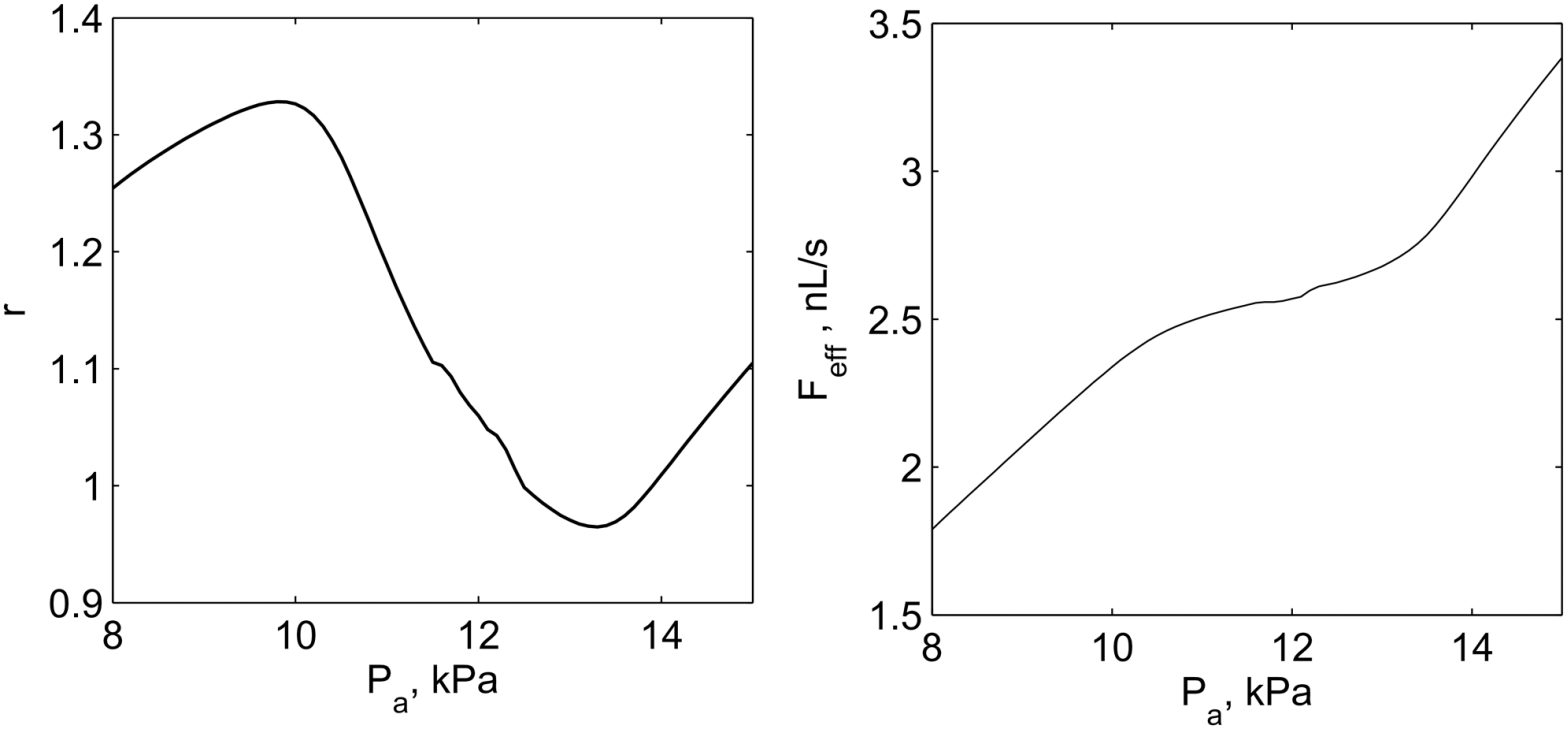
Autoregulation dynamics of the model. Relative response of afferent the arteriole radius *r* (left panel) and flow in the efferent arteriole *F*_*eff*_ (right panel) on increasing arterial pressure *P*_*a*_. Well pronounced regulation is observed within the range of 10–13 kPa.

Although the model is significantly simplified and does not contain a detailed description of all physiological mechanisms, its dynamical features include TGF oscillations, the response of afferent arteriole to increasing inlet pressure and, as a consequence, the autoregulation of efferent arteriolar blood flow (Fig 7).

## Results

### Topological properties of the network

We used the algorithms described above to construct models of both the renal vascular tree and a simple asymmetric bifurcating tree.

**Fig 8:**
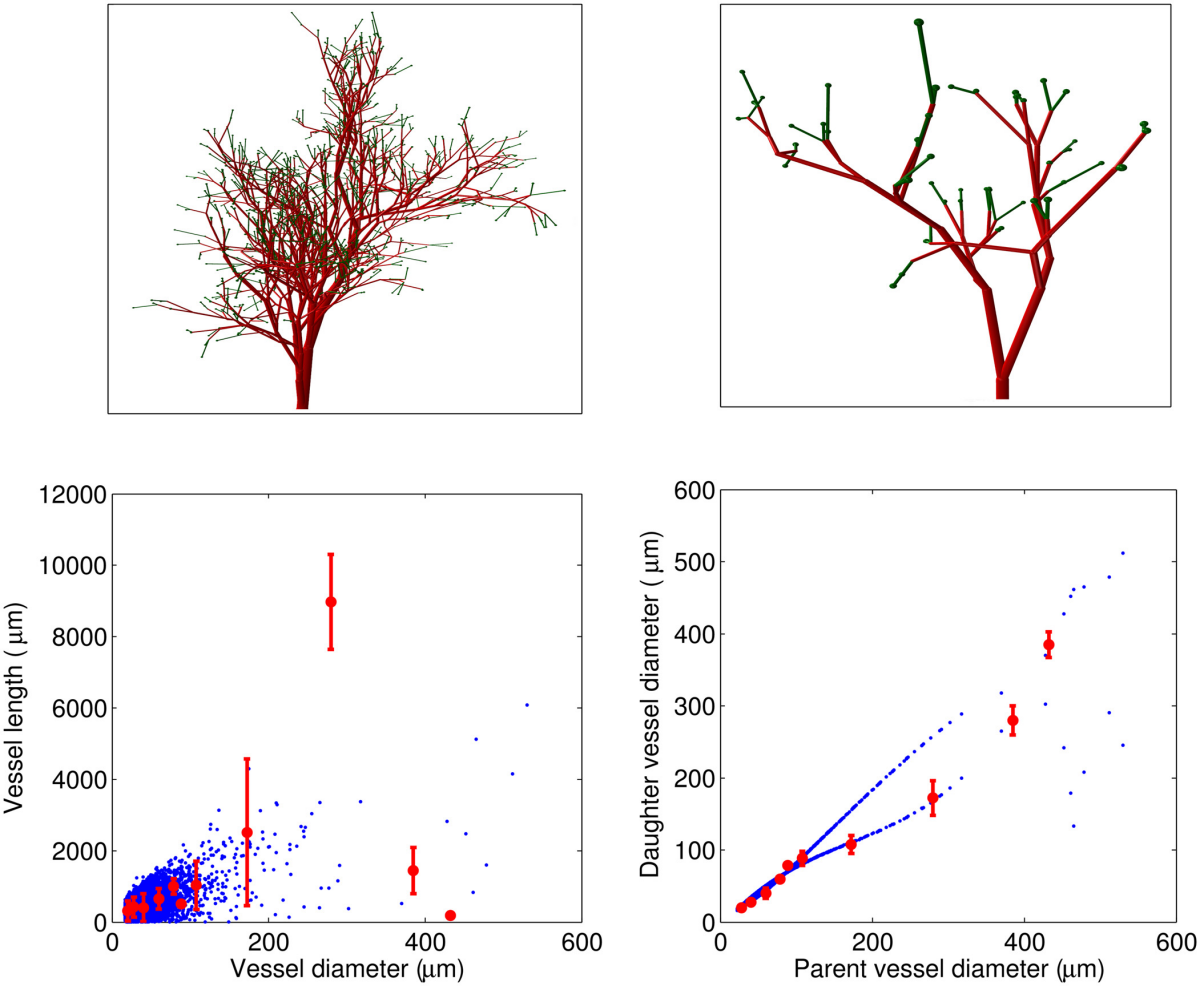
Simulated vascular structure based on the data published by Nordsletten et al [15] with afferent arterioles only at the top of bifurcating tree (ABT). Top panel: 3D visualizations of the vascular trees with *D*_*stop*_ = 22*μ*m and *D*_*initial*_ = 200*μ*m and 70*μ*m (left and right panels, respectively). Bottom panel: Vessel length as a function of the vessel diameter (left) and daughter vessel diameter as a function of the parent vessel diameter (right) for simulations (blue) and data described in Ref [15] (red). Note that the difference between simulated and experimental results for the daughter vessel diameter is related to the fact that we use Murray’s law to calculate the diameter of the second daughter vessel.

*Simple asymmetric bifurcating tree*. Fig 8 shows an example of a simulation of the ABT structure based on the experimental data described by Nordsletten et al. [15]. The plots in the bottom panel show a high correlation of the simulated structure with the experimental data. Note, however, the divergence in the relation between daughter and parent vessel diameter in the simulated and the experimental data. As noted above, the experimental data are grouped according to Strahler order, and two daughter vessels will not necessarily belong to the same Strahler order. This is especially likely if the two daughter vessels have a large difference in diameter. In this case the larger daughter vessel will, on average, be expected to have a greater Strahler order than the one with the smaller diameter, since there is a greater likelihood for further downstream bifurcations in the branch coming from the larger daughter vessel. It is therefore not to be expected that the simulated and experimental distributions will be identical.

**Fig 9:**
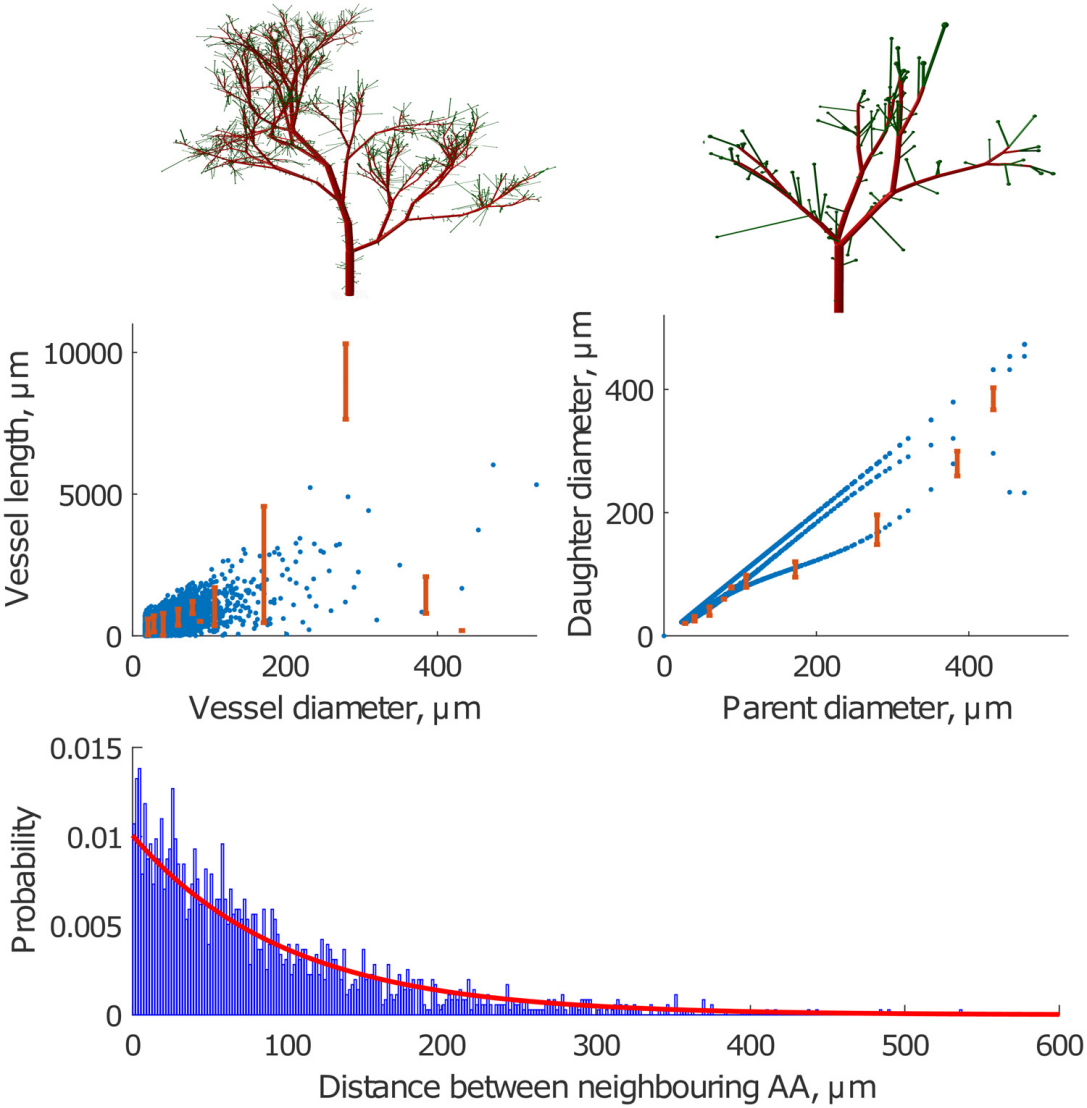
Simulated vascular structure with exponential distribution of afferent arterioles along the tree (KSABT). Top panel: 3D visualizations of vascular trees with *D*_*stop*_ = 22 *μ*m and *D*_*initial*_ = 200 *μ*m and 70 *μ*m (left and right panels, respectively). Middle panel: Vessel length as a function of the vessel diameter (left) and daughter vessel diameter as a function of the parent vessel diameter (afferent arterioles excluded from calculations)(right) for simulation (blue) and data described in Ref [15] (red). Bottom panel: Probability distribution of the distances between two neighboring afferent arterioles obtained from simulations (blue) and the approximation to the experimental data (red).

*Bifurcating tree with exponentially distributed afferent arterioles*. An example of a simulation of a KSABT structure is shown in Fig 9. Note that there is an even more pronounced difference in the dependence of daughter vessel diameter on parent vessel diameter compared to the experimental data [15]. The reason is that KSABT structure allows afferent arterioles to branch from any vessel (except the renal artery) and recalculates the vessel diameter after each bifurcation in accordance with Murray’s law. This algorithm leads to segmentation of large vessels so that at the end of each segment the vessel divides into an afferent arteriole and a segment with a slightly reduced diameter. The distribution of the distances between afferent arterioles (Fig 9, bottom panel) is based on the experimental data obtained from optical clearing methods [16, 17].

Notice that the number of afferent arterioles in the ABT and KSABT structures differ even though the parameters were similar in the two cases (see Table 1), and that in the KSABT structure the vessels feeding the afferent arterioles are, on average, wider than for the ABT structure where afferent arterioles appear only at the top of the tree. The larger diameter of the feeding vessels in the KSABT structure reduces the hemodymamic resistance of the vessels and, consequently, the pressure drop from the renal artery to afferent arterioles will be smaller when compared to that in the ABT structure.

**Table 1:** Example of statistical data on afferent arterioles in ABT and KSABT. Simulations were done at the same parameters *D*_*stop*_ = 22 *μ*m and *D*_*initial*_ = 530 *μ*m. RA denotes renal artery, AA denotes afferent arteriole.

Structure	Diameter RA, *μ*m	Number AA	Std. Dev.	Diameter AA, *μ*m	Std. Dev., *μ*m
ABT	530	20188	-	19.37	1.36
KSABT	530	32127	-	19.25	1.81

### Dynamical properties of the network

**Fig 10:**
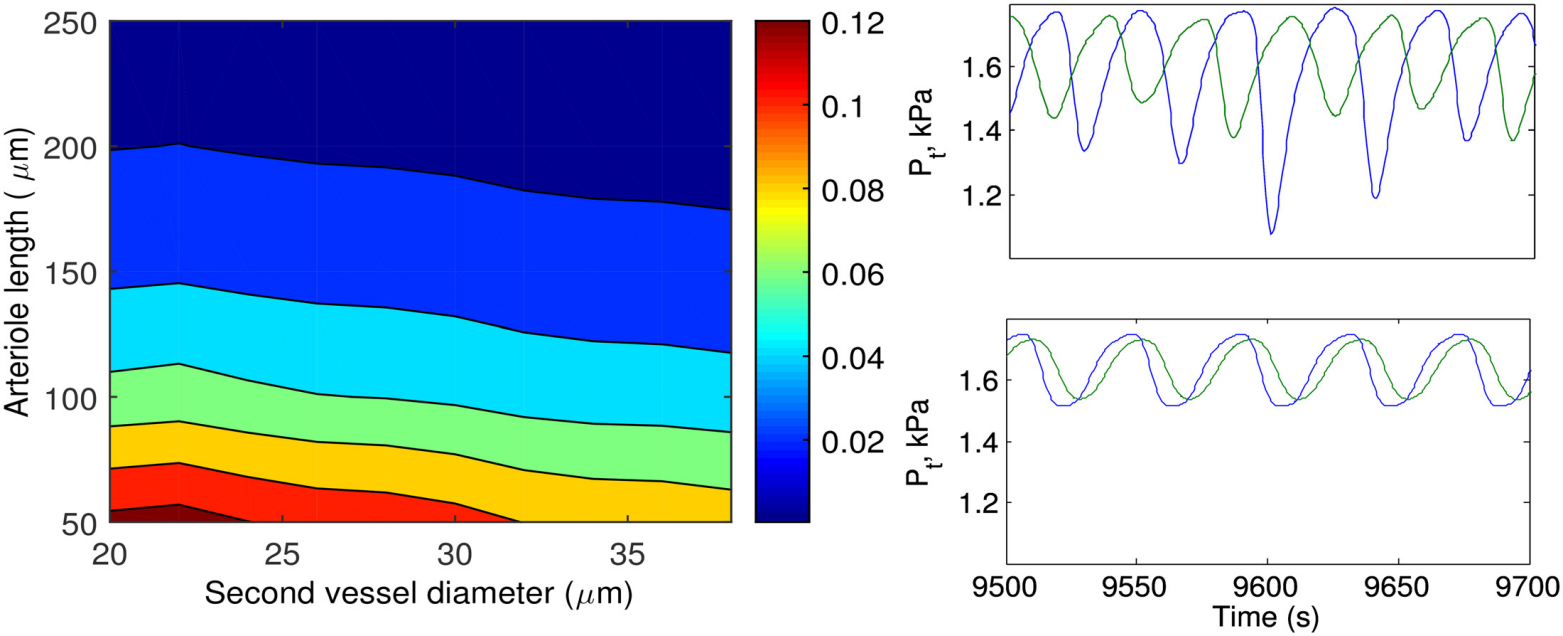
Effect of electrotonic interactions. Left panel: Color coded diagram of the strength of electrical signal propagation along afferent arteriole to the second daughter vessel. Simulations are performed for the vacular network without nephrons (test current was applied to the end of the first daughter vessel and measured at the end of the second daughter vessel). Right panel: Tubular pressure *P*_*t*_ variations of two neighboring nephrons with ≈0.05 (top) and ≈0.1 strength of electrical interaction (bottom).

As discussed above, nephrons can interact with each other via hemodynamic and/or electrical coupling. These interactions can lead to different dynamical patterns, and can affect the physiological properties of the whole kidney. The vascular structure is important for the characteristics of both types of interactions. It affects the electrical properties of the vascular tree in the following ways:

- The longer the distance between the sites where the electrical signal is generated and received, the weaker the signal. Hence, nephrons with long afferent arterioles and longer distances between each other show weaker interaction compared to nephrons connected via shorter distances (Fig 10) [39];
- At a branch point, the signal propagates better to the vessel with the larger diameter. This leads to weaker interaction between nephrons whose afferent arterioles originate from large vessels, while the strongest coupling is observed at the top of the tree, where the diameters of the daughter vessels are of similar scale.

To estimate the effect of vessel dimensions on the electrical coupling on a pair of nephrons, we performed simulations for a minimal branching structure consisting of one branch point and three vessels, a root vessel and two afferent arterioles. The lengths of the root vessel and the first afferent arteriole are fixed at 300 *μ*m and 50 *μ*m, respectively, and the diameter of the afferent arteriole is fixed at 20 *μ*m. The length and diameter of the second afferent arteriole are varied from 50 to 250 *μ*m and from 20 to 40 *μ*m, respectively. The root vessel diameter is adjusted according to Murray’s law. Fig 10 illustrates how the coupling strength depends on the length and diameter of the second afferent arteriole. Inspection of the figure shows that electrical interaction in a tree with short afferent arterioles leads to synchronization of nephron dynamics (right bottom panel), while in case of long afferent arterioles the nephrons are desynchronized (right top panel).

A characteristic feature of the renal vascular tree is that the pressure drop from the renal artery to the start of the afferent arteriole is small, whereas there is a relatively large pressure drop across the afferent arteriole [40]. This allows for efficient regulation of the glomerular filtration pressure through regulation of the resistance of the afferent arteriole.

**Fig 11:**
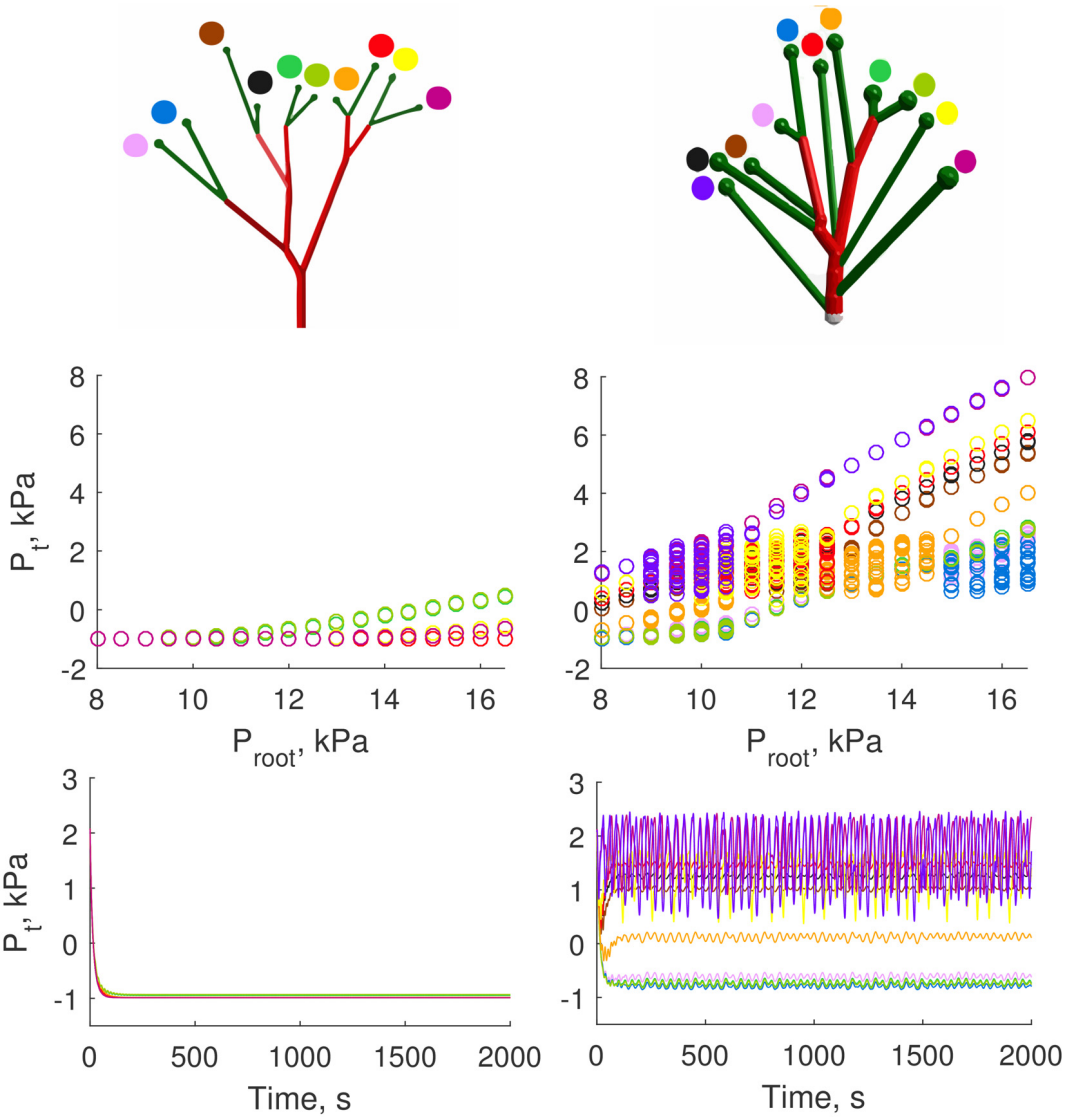
Simulation results for ABT (left) and KSABT (right) for *D*_*initial*_ = 40 *μ*m and *D*_*stop*_ = 22 *μ*m. Top panel: 3D visualization of simulated structures. Middle panel: Possible tubular pressure values for each nephron as function of the feeding pressure. For the ABT structure, the nephrons are inactive for the whole range of the root feeding pressure, while for the KSABT structure all nephrons are active within a wide range of the root feeding pressure. Bottom panel: Dynamics of tubular pressure *P*_*t*_ variations in all nephrons for pressure in the feeding vessel equal to 10 kPa (∼ 75 mmHg). This pressure level is insufficient for nephrons in the ABT structure to oscillate while for the KSABT structure the nephrons show oscillatory dynamics. Negative values of tubular pressure for the ABT structure are the consequence of insufficient pressure in the afferent arteriole to balance venous and filtration pressure and flow. Tubular pressure shown on the middle and bottom panels is color coded for each nephron according to the color markers on the top panel.

Simulations on the two types of vascular structures show that the vascular structure with exponentially distributed AAs (KSABT) has much lower pressure drop compared to the ABT. Simulations for a small branch of 40 *μ*m of initial diameter (Fig 11) with realistic hemodynamic resistances and root feeding pressure *P*_*root*_ clearly show that in the case of KSABT (right panel) the tubular pressure *P*_*t*_ of all nephrons is quite high and TGF oscillations can be observed. For the ABT, however, the root feeding pressure for the nephrons is low and outside the working range of the single nephron model that leads to negative values of *P*_*t*_ (left panel). In the nephron model that we use the negative values of tubular pressure are observed when the root feeding pressure is low and cannot balance flows and pressures on the venous side.

The higher pressures in the KSABT structure can be explained by two related factors: (i) the total number of branching levels is smaller in the KSABT than in the ABT network, and, (ii) the average diameter of feeding vessels is larger in KSABT, reducing the pressure drop between the renal artery and the afferent arterioles and providing higher pressure to the nephrons. The working pressure interval is depicted on the bottom panel of Fig 11. All nephrons in the KSABT work properly from 8 kPa in the root artery. This allows us to use a realistic pressure (around 13–13.5 kPa) in the renal artery. In the case of ABT there is no oscillatory behavior even at “hypertensive” values of the pressure.

**Fig 12:**
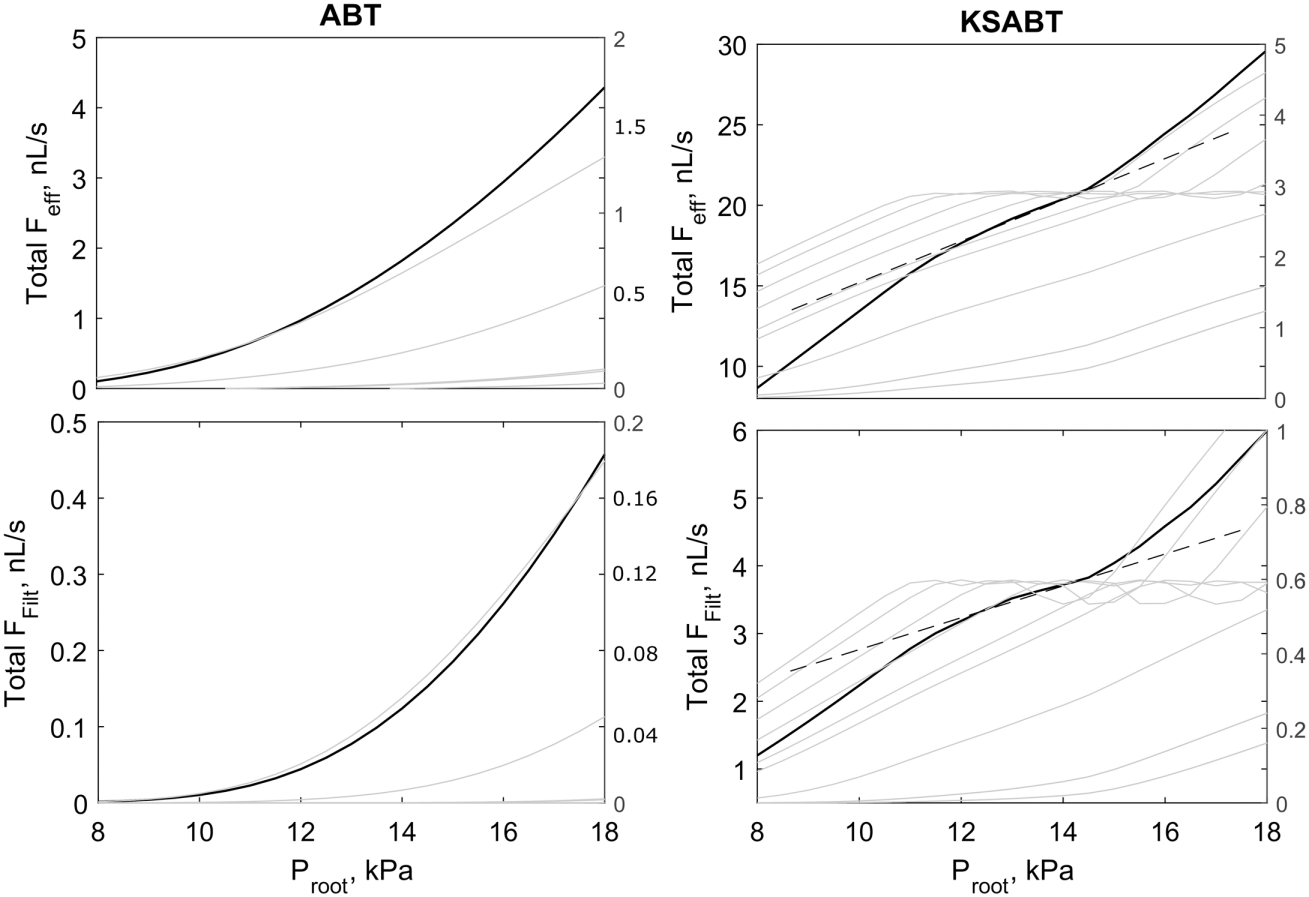
Comparison of net flow in efferent arterioles and glomerular filtration rate for ABT (left) and KSABT (right) structures shown in Fig 11. Top panel: Blood flow in efferent arterioles as a function of root pressure. Bottom panel: Glomerular filtration rate as a function of root pressure. Solid black curves (with left axes) show net blood flow and net glomerular filtration rate. Gray curves (with right axes) represent the same characteristics for individual nephro-vascular unit. Dashed black lines highlight the region of the strongest autoregulation.

Autoregulation properties of the two types of structures differ. Nephrons in ABT are perfused at inadequate pressure to support autoregulation of total efferent flow or glomerular filtration rate (Fig 12, left). In contrast, the structure of KSABT provides nephrons with a higher pressure, supporting autoregulation at the level of individual nephrons and net flow (Fig 12, right). A significant shift in nephron feed pressure in KSABT leads to prolonged and smoothed range of autoregulation for net flows.

## Discussion

The systemic circulatory system plays a central role in supplying organs and tissues with oxygen and nutrients and removing metabolic waste products. On a large scale, the circulatory system can be viewed as a branching network where larger vessels on the arterial side branch into smaller and smaller vessels until the capillary level is reached. It is clear however that there are large differences in the structure of the vascular system among different tissues and organs, and that this difference relates to the specific functions of the tissue and organ. In other words, the vascular topology is optimized to subserve the specific needs of a given organ or tissue. In skeletal muscle, the main need is to allow for large variations in total blood flow so as to match the supply with demand during muscle work. In other organs, like the brain, there is no need for large variations in overall flow, but there is a need to redirect flow to local areas where the metabolic activity is high. In the kidney, the critical aspect of the circulation is not only to supply the tissues with oxygen and nutrients, but rather to support the filtration of plasma at the glomerulus of each nephron. For example, in the human kidneys an amount of fluid corresponding to approximately 3 times the body weight is filtered every 24 hours. This difference in function poses restrictions on the design of the vascular system in the specific organs and tissues. Many diseases, e.g. hypertension and diabetes, affect the vascular system, and the morbidity and mortality of these diseases are to a large extent associated with the diseases’ effects on the vascular system. The effect of the disease in a given organ is not only the result of the disease process itself, but also depends on the specific topology of the vascular system in the organ [10].

In recent years several new imaging modalities, e.g. *μ*CT, and confocal microscopy, have made it possible to obtain images of the entire microcirculation in a given organ [9, 15]. This has paved the way for a better understanding of the complex interrelationship between organ function and vascular topology. To obtain a thorough understanding of the role of vascular topology in organ function it is necessary to combine experimental and modeling approaches, since many questions cannot be addressed experimentally. In this connection, a major task is to create realistic models of the vascular tree in a given organ as a basis for further model studies.

In this study we present new experimental findings of the 3D arterial and afferent arteriolar structure and we develop a probability-based algorithm for generating such structures from the data.To study the role of kidney specific vascular structure we also introduce a mathematical model that describes blood flow dynamics and nephron-to-nephron interactions in the arterio-nephron network. The simulation results indicate that the arterial structure in the kidney minimizes the pressure drop between the main renal artery and glomeruli, and it affects nephron-nephron interactions.

One of the main results is that the distances between individual AAs are exponentially distributed. This conclusion is the result of the analysis of 3D data sets with more than 150 afferent arterioles obtained with the optical clearance method. This result differs from that in most other organs where the interbranch distance seems to follow a lognormal distribution [31, 32]. Exponential distributions are typically seen in Poisson processes where the time or distance between events are independent of each other. The genetic/molecular mechanisms that underlies such a branching pattern are unknown, and an interesting subject for further theoretical work. Statistical distributions derived from our data and from the literature [15] form the basis for our structure generating algorithm.

The microcirculation of the kidney is unusual because it has 2 capillary networks, the glomerular capillaries and the peritubular capillaries, connected through the efferent arteriole. Compared to capillary networks in other organs, the glomerular capillaries operate with high intravascular pressure, a condition required to to drive the filtration of fluid across the capillary wall into the lumen of the proximal tubule. The high filtration rate is a prerequisite for the ability of the kidneys to regulate the volume and composition of the extracellular fluid. When we used a simple bifurcating tree (the ABT algorithm), it was apparent that with the vessel dimensions reported by Norsletten et al. [15], the pressure drop from the renal artery to the glomerular capillaries exceeds the value found experimentally [40]. This is not surprising, since in the ABT the AAs only appear at the terminal branch points of the tree, an assumption that maximizes the hemodynamic resistance between the renal artery and the glomerulus. In contrast, when afferent arterioles are distributed exponentially across the vascular tree and allowed to branch from any arterial segment, the resulting pressure in the glomerular capillaries is significantly greater, and in a range that is compatible with normal nephron function.

It is of interest to note that only the KSABT algorithm resulted in a realistic number of nephrons for the whole kidney (Table 1), i.e. around 33,000 [41, 42]. Because the ABT only has glomeruli at the terminal branches, the number of nephrons becomes much lower than in the KSABT, where AAs, and thus nephrons, also originates from the larger vessels. For the ABT to give a realistic number of nephrons, it will be necessary to start it with a vessel of an unrealistically large diameter.

The topology of the arterial network influences not only the pressure drop along the network, but also electrically mediated nephron-nephron interactions. As shown in Fig 10 signal propagation depends on the dimensions of the branching vessels. A signal from the macula densa is conducted through the nephron’s afferent arteriole and, at the next bifurcation, propagates preferentially to the largest diameter branch. A nephron whose AA originates from a larger vessel will therefore be less efficient in synchronizing with its neighbors than will a nephron at the distal end of the arterial tree, where the vessels tend to be of a similar scale. Previous studies on nephron-nephron interactions have used a simple bifurcating tree when investigating the network properties of the renal circulation [23, 24]. Such simplifications, by ignoring significant structural asymmetries, could therefore overestimate synchronization. Another important feature is that asymmetrical connections of endothelial cells can lead to anisotropic propagation of electrical signal between two afferent arterioles and can affect nephron-to-nephron interaction.

Although our algorithm results in architecturally realistic arterial networks for the renal circulation, the tubular pressure distribution between nephrons is exaggerated, cf. Fig 11. Experimental data has shown that the proximal tubular pressure is quite uniform in nephrons on the surface of the kidney [43]. This suggests the presence of additional processes that acts to homogenize the pressure distribution between nephrons. One possibility is that the length, and thereby the resistance, of the AA may vary according to the branch site, i.e. if the AA branches from a larger vessel with a high intravascular pressure it may be longer compared to a similar vessel that from a smaller vessel. At present, there is no data on renal vascular morphology to test this possibility, and it has therefore not been included in the present algorithm. Another possibility is that the individual vessels actively adjust their radius to compensate for differences in the feeding pressures. The AA has a myogenic response [44] that allows it to adjust its radius in response to changes in the intravascular pressure. An increase in pressure will provoke vasoconstriction, increasing the hemodynamic resistance of vessel, reducing glomerular pressure towards the control value. This mechanism plays an important role in renal autoregulation of blood flow [45] and is implemented in our model in a simplified way.

An anatomical feature not explicitly included in the present algorithm is the presence of triplets and quadruplets at the branch sites, as reported in [30]. Such triplets or quadruplets of AAs mostly occur at the top of the tree, since the small arteries will always split onto two AAs. We do not explicitly model the population of juxtamedullary nephrons in the present work. However, it is known that this subpopulation of nephrons predominantly derive their afferent arterioles from the larger vessels in the arterial network, e.g. the arcuate and subarcuate arteries [28]. However, the available data does not allow specific modeling of this subpopulation.

In the present work we have chosen to use a simple nephron model [38]. The model, detailed below, has primitive representations of TGF and the afferent arteriole that cause a single combined effect on afferent arteriolar diameter in response to arterial pressure change. When several copies are combined in a network configuration the model generates interactions [20, 21, 23] similar to those produced with more detailed representations of TGF and the afferent arteriole [24]. Because of the complexity of the network a minimal model such as the one we use here best serves the purposes of the study by providing the clearest opportunity for understanding the contributions of the vascular structure. To test stability of the model we ran simulations for different values of parameters *C*_*hdr*_(0.3–5*nL*/*kPa*), *β*(0.4–0.67), *α*(12–20). We found minor quantitative changes but the dynamics of the model with ABT and KSABT structures remained qualitatively unchanged.

In conclusion, we have demonstrated that the renal vasculature has specific characteristics that were not taken into account in previous modeling studies. We have developed a new algorithm for generating a renal arterial network using experimental data on the length and radius distribution of renal vessels. The resultant network topology closely resembles that found in normal rats. We have found that in the contrast to the simpler known algorithm, the kidney specific vascular tree (based on our data and from the literature) contained a realistic number of nephrons, displayed adequate pressure levels in the nephrons, and tended to weaken interactions between nephrons whose afferent arterioles originated from larger vessels compared to the nephrons on top of the tree.

## Materials and Methods

### Experiment

**Fig 13:**
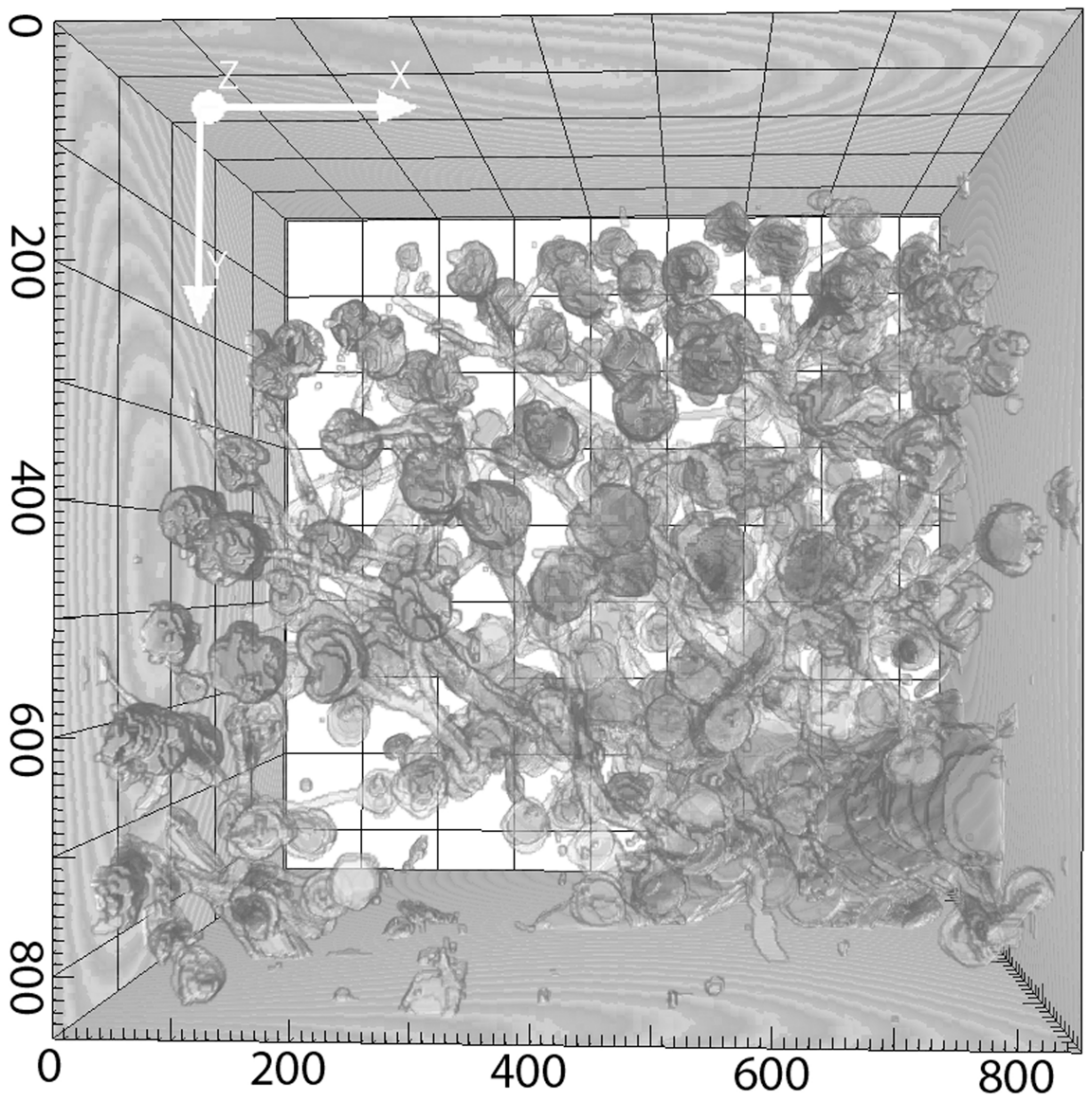
The image shows the full kidney stack after reconstruction. The dimensions of the stack are 850 x 850 x 1018 *μm*. The axis labels represent the original coordinates in *μm*, but note that the reconstructed data has shrunk about 20% in all dimensions compared to living tissue.

All experimental protocols were approved by the Danish National Animal Experiments Inspectorate and were conducted in accordance with guidelines of the American Physiological Society. A 12 week old Sprague-Dawley rat was anesthetized (intraperitoneal injection of pentobarbital), a catheter was inserted into the aorta, and the vena cava was opened. The blood was removed by perfusing the animal with PBS with nifedipine and HEPES to prevent blood clotting. Thereafter the animal was perfused with biotin (2 mg/ml) that binds to endothelial cells, washed with PBS and perfused with Alexa-647 Streptavidin (20 mg/ml). The animal was then fixed with 4% paraformaldehyde in PBS using a pressure of 90 mmHg. The kidneys were removed and a section of one of the kidneys was clarified according to Erturk et al. [17], using dehydration steps in THF and final clearing in DBE [16]. Tissue dehydrated by this method shrinks approximately 20% in each dimension. The kidney slice was scanned with a 633 nm laser in a Zeiss LSM 710 equipped with a 10X/0.3 NA objective. Two 3D stacks were recorded, covering 850 x 850 x 497 *μm* and 850 x 850 x 1018 *μm*. The second image stack was obtained with spectral imaging to obtain both green autofluorescence from tubules and the Alexa-633 signal from both vessels and tubuli. The combined image showing tubuli in one channel and vessels and tubuli in the other was used as input for the segmentation protocol. The dual channel image was then automatically segmented in the open source image processing package “FiJi”, using pixel-based segmentation plugin “Trainable Weka Segmentation”. After segmentation the 150 images in the stack were manually corrected in FiJi for missing features and holes in vessels. The 3D representations (Figs 3 and 13) were performed with open platform for bioimage informatics “Icy”.

### Computational implementation

To build a vascular structure using the present algorithm four distributions are needed: *G*_*ddp*_ (daughter-parent diameter distribution), *G*_*vlvd*_ (vessel length–vessel diameter distribution), *E*_*aad*_ (distribution of distances between two neighboring AAs) and *G*_*aad*_ (distribution for afferent arterioles diameter), where *G*_*ddp*_, *G*_*vlvd*_ and *G*_*aad*_ are assumed to be Gaussian distributions and *E*_*aad*_ is an exponential distribution. All distributions were obtained from experimental data. Two parameters are used to control the structure size: *D*_*initial*_ describes the diameter of the first vessel and *D*_*stop*_ is the vessel diameter where bifurcations stop.

**Fig 14:**
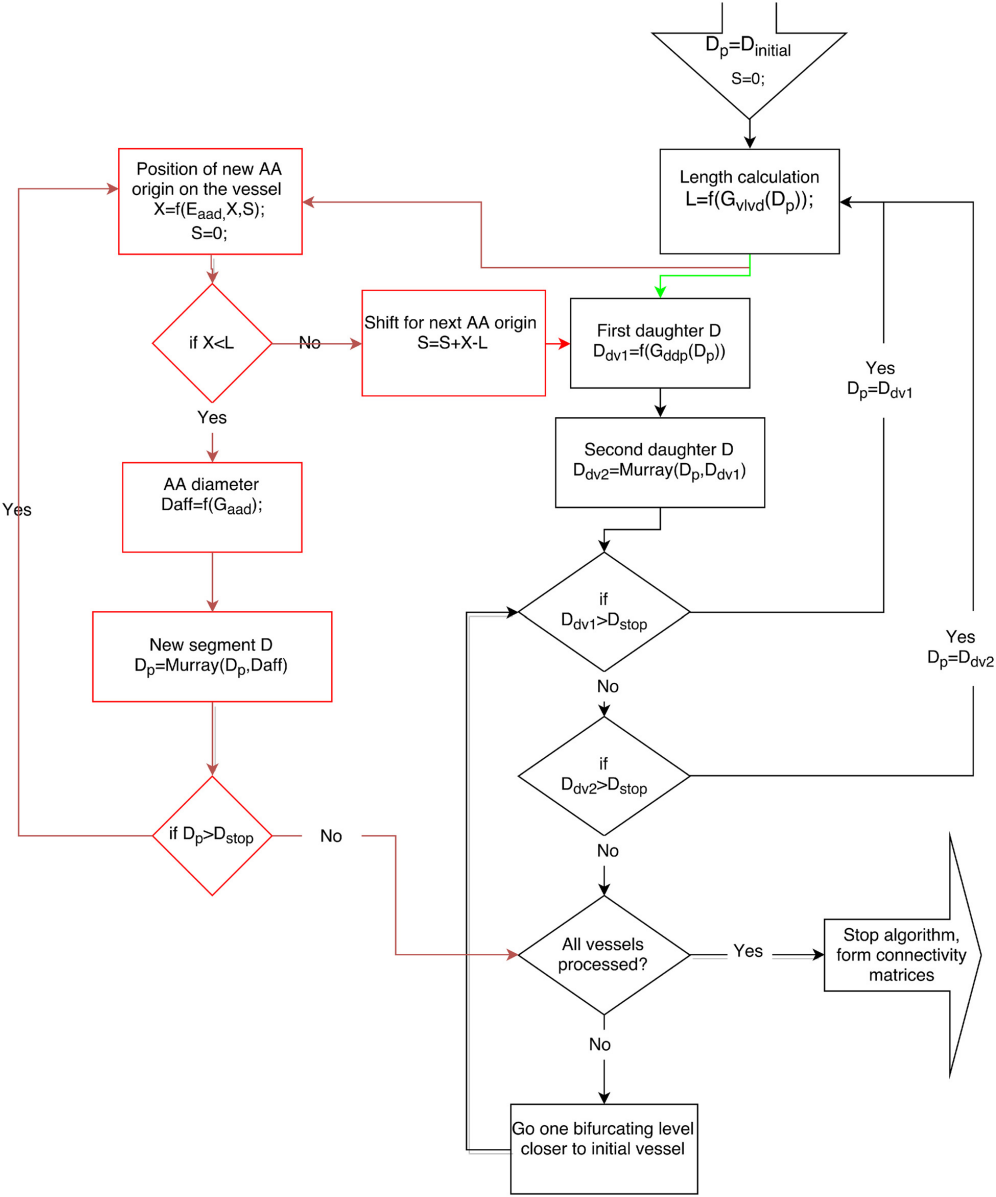
Functional diagram of the algorithm. Black arrows show common steps to create both structures. Green arrows are the main steps of recursively bifurcating algorithm for creation of ABT. Red arrows are the main steps for recursively bifurcating algorithm with additional segmentation for KSABT. *D*_*initial*_ and *D*_*stop*_ are parameters; *G*_*ddp*_, *G*_*vlvd*_, *G*_*aad*_, and *E*_*aad*_ are the distributions obtained from experimental data; *D*_*p*_, *D*_*dv*1_, *D*_*dv*2_ are the diameter of current segment of the parent vessel, and the first and second daughter vessel diameters; *L* is the length of the current vessel; *X* is the position of AA origin along the vessel; *S* is a shift of AA position that accounts for the distance to the neighboring AA located on the other (e.g. parent) vessel; *D*_*aff*_ denotes the diameter of the afferent arteriole.

This algorithm combines a recursively bifurcating algorithm (for a simple bifurcating structure) and recursively bifurcating algorithm with additional segmentation (for a structure with exponentially distributed distances between AAs) (Fig 14).

**Simple recursively bifurcating algorithm**
1. Calculate the length of the first vessel using *D*_*initial*_ and *G*_*vlvd*_ distribution;
2. Calculate the diameter of the first daughter vessel *D*_*dv*1_ using *G*_*ddp*_;
3. Calculate the second daughter diameter *D*_*dv*2_ using the parent diameter, the first daughter diameter and Murray’s law;
4. If the first daughter diameter is bigger than *D*_*stop*_, consider the first daughter vessel as a new parent vessel;
5. Repeat steps 2, 3, 4 until *D*_*dv*1_ becomes less than or equal to *D*_*stop*_;
6. If *D*_*dv*2_ is larger than *D*_*stop*_, consider this daughter vessel as a new parent vessel. If it is less, go one level below the bifurcation and repeat step 6;
7. Repeat steps 2, 3, 4, 5, 6 until there are no vessels whose diameter is larger than *D*_*stop*_ and without daughter.

**Additional segmentation**
1. Find a position of the next AA branching using *E*_*aad*_ and the length of the current vessel.
2. If the previous AA branches from the other vessel (e.g., parent vessel), use a shift value (i.e., accumulated distances to AA on the other vessel). The found position denotes the end of the current segment. If the calculated position is not on the current vessel, update the shift value and stop branching of this vessel, otherwise continue;
3. Add AA to the end of the segment with the diameter based on *G*_*aad*_;
4. Calculate the diameter of the next segment using Murray’s law, segment diameter and AA diameter;
5. If the next segment diameter becomes smaller than *D*_*stop*_, stop branching of this vessel.

### Description of electrical coupling

We simplified the model described in Ref. [46] with some additional assumptions:

1. All related processes in the endothelium are much faster than the TGF feedback and, thus, all membrane potentials are instantly adjusted to the signal produced by the *macula densa*;
2. We use a linear representation of the whole-cell conductance. This allows us to calculate a matrix of connectivity coefficients between nephrons, instead of running an additional integration loop for each integration step of the whole model;
3. At the branch points, cells from each vessel split in numbers according to the diameters of the other two vessels. The conduction of such a connection is proportional to the number of connected cells from both sides;
4. We neglect latitudinal variability of endothelial membrane potentials, but still take into account longitudinal discreteness of the vascular bed.

This allows us to build a computationally simple, but physiologically relevant, model for the propagation of the electrical signals generated by the nephrons.

**Fig 15:**
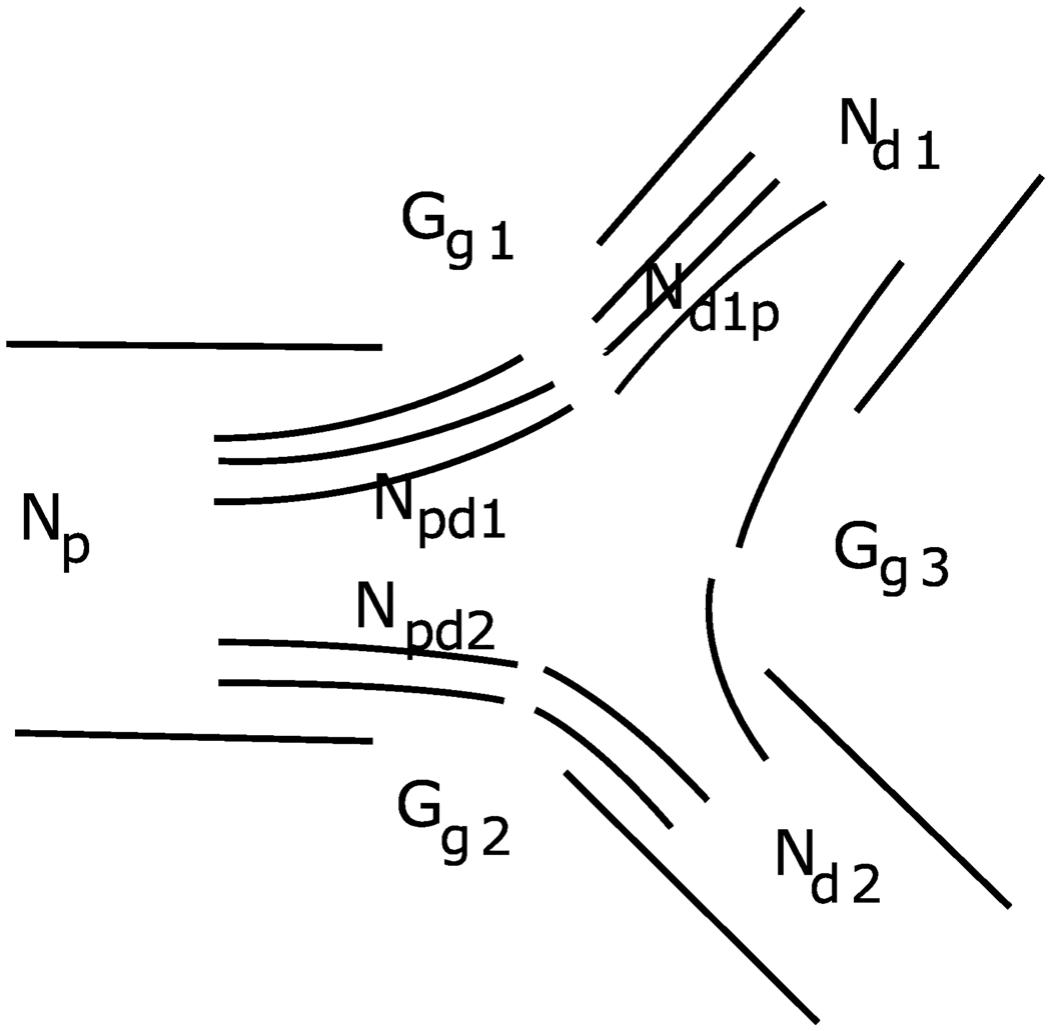
Schematic presentation of electrical coupling. A cross-section of a three vessel segment at the branch point with *N* endothelial cells and gap junction conductances *G*_*g*_.

Fig 15 represents the main notations of the coupling. Each endothelial cell is described by the whole-cell conductance *G*_*c*_ which can be linear or nonlinear, and instantaneous (very fast activated) or inertial (governed by the dynamics of gating variables). We use the simplest representation in the form of an instantaneous and linear whole-cell conductance *G*_*c*_ (assumptions 1 and 2) being connected in parallel with the whole-cell capacitance *C*_*c*_ and coupled with neighbors by means of gap junction conductances *G*_*gj*_.

There is considerable variability with regard to the reported morphological and electrical parameters of endothelial cells. For the present work we chose parameters to be in accordance with those in Ref. [46]. We assume that a vessel segment of diameter *D* contains *N* = *πD*/*W*_*c*_ endothelial cells in its cross-section, where *W*_*c*_ ≈ 5*μm* is a typical width of an individual cell. Similarly, we assume that the length *L* of the vessel segment is composed of *M* = *L*/*L*_*c*_ endothelial cells, where *L*_*c*_ ≈ 50*μm* is a typical effective length of an endothelial cell. The length of an endothelial cell is around 100 *μ*m. However, due to overlap of the ECs in the vessel wall, the effective length is approximately half the the real length of the cell [47].

We ignore possible spatial inhomogeneity of voltage distribution in a vessel cross-section. Thus, we describe each unit (piece of vessel with length *L*_*c*_) with its unit conductance *G*_*u*_ = *NG*_*c*_, unit capacitance *C*_*u*_ = *NC*_*c*_, and total gap junction conductance *G*_*g*_ = *NG*_*gj*_. A vessel segment is represented by a chain of *M* such coupled units.

At the branch (bifurcation) point three vessel segments are connected to each other. At this point we assume a triangle-shaped coupling geometry as shown in Fig 15. To calculate *G*_*g*1_, *G*_*g*2_, *G*_*g*3_ we use assumption 3 listed above. Cells from the *i*-th segment connected to the *j*-th and *k*-th segments are split in two parts according to circumference (or equivalently—diameters) of the *j*-th and *k*-th segments. So, *N*_*i*_ = *N*_*j*_ + *N*_*k*_, where *N*_*j*_/*N*_*k*_ = *D*_*j*_/*D*_*k*_ and *N* is the number of cells which is always an integer. However, this rule gives different number of cells for different vessels at the same branch point. For example, the number of cells *N*_*j*−*k*_ from *j*-th to *k*-th segment will differ from the number of cells *N*_*k*−*j*_ from the *k*-th to *j*-th segment. The existence and value of this mismatch can not be predicted in advance. Thus, we assume that the number of gap junctions between two segments is equal to the arithmetic average between the number of cells in the connected vessels. In this way we have some intermediate, but symmetric value for inter-segment coupled cells: *N*_*jk*_ = *N*_*kj*_ = (*N*_*j*−*k*_ + *N*_*k*−*j*_)/2. The conductance of such coupling is *G*_*g*_*jk*__ = *G*_*g*_*kj*__ = *N*_*jk*_*G*_*gj*_.

At the root of the tree the same rule for the conductance is applied. However, we assume that the diameter of vessels downstream from the root is large enough, so that each endothelial cell from the root segment will be connected to two endothelial cells from downstream vessels. This gives us *G*_*g*_*root*__ = 2*N*_*i*_*G*_*gj*_ for each connected vessel, where *N*_*i*_ is the number of cells in the circumference of the root segment.

### Description of nephron model

Physiological justification for all equations and expressions are given in Ref [38]. Here we focus on computational implementation of the model.

*P*_*t*_, *r*, *v*_*r*_, *X*_1_, *X*_2_, *X*_3_ denote dynamical variables of the system.
*F*_*Hen*_, *F*_*Filt*_, *P*_*g*_, *P*_*av*_, *P*_*eq*_, *P*_*el*_, *P*_*act*_, Ψ, *R*_*a*_, *R*, *C*_*e*_ are functions that have physiological meaning and are needed to calculate derivatives.
  *A*, *B*, *C*, *D* are coefficients of the 3rd order polynomial equation for the efferent plasma protein concentration, *C*_*e*_.
*P*_*v*_, *P*_*d*_, *R*_*a*0_, *R*_*e*_, *R*_*Hen*_, *C*_*tub*_, *H*_*a*_, *F*_*reab*_, *F*_*Hen*0_, *C*_*a*_, *a*, *b*, *ω*, *K*, *β*, Ψ_*max*_, Ψ_*min*_, Ψ_*eq*_ are constants.
*P*_*a*_, *T*, *α* are control parameters. Full list of parameters can be found in Table 2.

**Table 2:** Table of constants and parameters.

Parameter	Meaning	Value
*P*_*root*_	Arterial pressure in the root of the tree [*kPa*]	13.3
*C*_*tub*_	Compliance of the proximal tubule [*nL*/*kPa*]	3.0
*H*_*a*_	Arterial hematocrit	0.5
*P*_*v*_	Efferent arterial pressure [*kPa*]	1.3
*P*_*d*_	Distal tubular hydrostatic pressure [*kPa*]	0.6
*F*_*Hen*0_	Henle’s loop equilibrium flow [*nL*/*s*]	0.2
*F*_*reab*_	Proximal tubular reabsorption rate [*nL*/*s*]	0.3
*R*_*Hen*_	Hydrodynamic resistance of the loop of Henle [*kPa* × *s*/*nL*]	5.3
*R*_*a*0_	Afferent arteriolar equilibrium resistance [*kPa* × *s*/*nL*]	2.4
*R*_*e*_	Efferent arteriolar resistance [*kPa* × *s*/*nL*]	1.9
*ω*	Frequency coefficient of the damped oscillator [*kPa* × *s*^2^]	20.0
*K*	Damping coefficient [1/*s*]	0.04
*β*	Non-variable fraction of afferent arteriolar resistance	0.67
Ψ_*min*_	Lower activation limit of TGF	0.20
Ψ_*max*_	Upper activation limit of TGF	0.44
Ψ_*eq*_	Equilibrium activation	0.38
*C*_*a*_	Afferent plasma protein concentration [*g*/*L*]	54.0
*a*	Protein concentration parameter [*kPa* × *L*/*g*]	21.7*e*−3
*b*	Protein concentration parameter [*kPa* × *L*^2^/*g*^2^]	0.39*e*−3
*T*	Transport delay in the loop of Henle [*s*]	13.5
*α*	Tubuloglomerular feedback amplification	20.0
*L*_*c*_	Length of endothelial cell [*μm*]	50
*W*_*c*_	Width of endothelial cell [*μm*]	5
*G*_*gj*_	Gap junction conductance of endothelial cell [1/*MOhm*]	1/3
*G*_*c*_	Conductance of endothelial cell [1/*MOhm*]	1/8
*C*_*c*_	Endothelial cell capacitance [*pF*]	20
*V*_*rest*_	Cell membrane resting potential [*mV*]	−40
*C*_*hdr*_	Compliance of blood vessels [*nL*/*kPa*]	3.0

Example of initial condition is: *P*_*t*_ = 1.7923 kPa, *r* = 1.0221, *v*_*r*_ = −0.0149, *X*_1_ = 0.9177, *X*_2_ = 0.8903, and *X*_3_ = 0.8695.

The functions that are used in the model are described below.

Flow in the loop of Henle:

$$F_{Hen}=\frac{P_{t}-P_{d}}{R_{Hen}}$$ (14)

Preglomerular resistance

$$R_{a}=R_{a0}\times \left(\beta +\frac{1-\beta }{r^{4}}\right)$$ (15)

$$R=\frac{R_{a0}}{R_{a}}$$ (16)

We solve the third-order equation $AC_{e}^{3}+BC_{e}^{2}+CC_{e}+D=0$ to find *C*_*e*_, the efferent arteriolar plasma protein concentration. For appropriate parameter valse the equation has a single, positive solution. This solution is determined numerically for each integration step. *A*, *B*, *C*, *D* are found from the model equations:

$$A=b+R\times b\times H_{a}$$ (17)

$$B=a+R\times b\times C_{a}\times \left(1-H_{a}\right)+R\times a\times H_{a}$$ (18)

$$C=P_{t}-P_{v}+R\times a\times C_{a}\times \left(1-H_{a}\right)+R\times \left(P_{t}-P_{a}\right)\times H_{a}$$ (19)

$$D=\left(P_{t}-P_{a}\right)\times R\times C_{a}\times \left(1-H_{a}\right)$$ (20)

Functions of plasma protein concentrations:

$$P_{g}=b\times C_{e}^{2}+a\times C_{e}+P_{t}$$ (21)

$$P_{av}=\frac{1}{2}\times \left(P_{a}-\left(P_{a}-P_{g}\right)\times \beta \times \frac{R_{a0}}{R_{a}}+P_{g}\right)$$ (22)

$$F_{Filt}=\left(1-H_{a}\right)\times \left(1-\frac{C_{a}}{C_{e}}\right)\times \frac{P_{a}-P_{g}}{R_{a}}$$ (23)

The tubuloglomerular feedback function Ψ:

$$\Psi =\Psi_{max}-\frac{\Psi_{max}-\Psi_{min}}{1+\frac{\Psi_{eq}-\Psi_{min}}{\Psi_{max}-\Psi_{eq}}\times exp\left(\alpha \times \left(\frac{3\times X_{3}}{T\times F_{Hen0}}-1\right)\right)}$$ (24)

The depolarisation of the cells in the afferent arteriole due to the TGF signal from the *macula densa* is assumed to be directly proportional to Ψ.

Equilibrium pressure:

$$P_{el}=1.6\times \left(r-1\right)+2.4\times e^{\left(10\times (r-1.4)\right)}$$ (25)

$$P_{act}=\frac{4.7}{1+e^{\left(13\times (0.4-r)\right)}}+7.2\times r+6.3$$ (26)

$$P_{eq}=P_{el}+\Psi \times P_{act}$$ (27)

Note that the parameter *P*_*a*_ in the single nephron-model becomes a variable in the nephro-arterial network.
